# Effects of obesogenic diet and 17β-estradiol in female mice with *APOE* 3/3, 3/4, and 4/4 genotypes

**DOI:** 10.3389/fnagi.2024.1415072

**Published:** 2024-09-13

**Authors:** Amy Christensen, Cassandra J. McGill, Wenjie Qian, Christian J. Pike

**Affiliations:** Davis School of Gerontology, University of Southern California, Los Angeles, CA, United States

**Keywords:** *APOE*, estrogen, metabolic impairment, lipidomics, microglia, obesity

## Abstract

The main genetic risk factor for Alzheimer’s disease (AD) is the apolipoprotein E ε4 allele (*APOE4*). AD risk associated with *APOE4* disproportionately affects women. Furthermore, human and rodent studies indicate that the cognitive deficits associated with *APOE4* are greater in females. One modifiable AD risk factor is obesity during middle age. Given that approximately two-thirds of US adults are overweight, it is important to understand how obesity affects AD risk, how it interacts with *APOE4*, and the extent to which its detrimental effects can be mitigated with therapeutics. One intervention study for women is estrogen-based hormone therapy, which can exert numerous health benefits when administered in early middle age. No experimental studies have examined the interactions among *APOE4*, obesity, and hormone therapy in aging females. To begin to explore these issues, we considered how obesity outcomes are affected by treatment with estradiol at the onset of middle age in female mice with human *APOE3* and *APOE4*. Furthermore, to explore how gene dosage affects outcomes, we compared mice homozygous for *APOE3* (3/3) and homozygous (4/4) or hemizygous (3/4) for *APOE4*. Mice were examined over a 4-month period that spans the transition into reproductive senescence, a normal age-related change that models many aspects of human perimenopause. Beginning at 5 months of age, mice were maintained on a control diet (10% fat) or high-fat diet (HFD; 60% fat). After 8 weeks, by which time obesity was present in all HFD groups, mice were implanted with an estradiol or vehicle capsule that was maintained for the final 8 weeks. Animals were assessed on a range of metabolic and neural measures. Overall, *APOE4* was associated with poorer metabolic function and cognitive performance. However, an obesogenic diet induced relatively greater impairments in metabolic function and cognitive performance in *APOE3/3* mice. Estradiol treatment improved metabolic and cognitive outcomes across all HFD groups, with *APOE4/4* generally exhibiting the greatest benefit. *APOE3/4* mice were intermediate to the homozygous genotypes on many measures but also exhibited unique profiles. Together, these findings highlight the importance of the *APOE* genotype as a modulator of the risks associated with obesity and the beneficial outcomes of estradiol.

## 1 Introduction

The ε4 allele of apolipoprotein 4 (*APOE4*) is the most significant genetic risk factor for late-onset Alzheimer’s disease (AD). The mechanisms by which *APOE4* impacts AD risk are thought to be numerous and include regulation of β-amyloid clearance, metabolism, inflammation, glial dynamics, and vascular function (Serrano-Pozo et al., 2021; Parhizkar and Holtzman, 2022; Tzioras et al., 2019; Norwitz et al., 2021). The multifactorial nature of *APOE4*-associated risks for age-related cognitive decline and AD has been hypothesized to result from apoE’s actions on cell types and systems throughout the body (Martens et al., 2022). Consistent with its widespread impacts, *APOE4*-associated AD risk is thought to be influenced by its interaction with other risk factors, including obesity (Jones and Rebeck, 2018), female sex (Valencia-Olvera et al., 2023), and even the combined effects of obesity and sex (Moser and Pike, 2016).

Obesity at midlife is a risk factor for AD and related disorders as well as age-related cognitive decline (Whitmer et al., 2005; Besser et al., 2014). The relationship between obesity and neural vulnerability is modulated by the *APOE* genotype (Zade et al., 2013), which aligns with the findings that *APOE4* carriers are at increased risk for metabolic syndrome and cardiovascular disease (El-Lebedy et al., 2016; Torres-Perez et al., 2016). However, the relationship is complicated by interactions that are incompletely understood (Moser and Pike, 2016). In studies of both humans (Mole et al., 2020; Osiecka et al., 2023; Shinohara et al., 2020; Zhao et al., 2023; Coad et al., 2022) and rodents (Moser and Pike, 2017; Arbones-Mainar et al., 2016; Johnson et al., 2017; Jones et al., 2021; Pandit et al., 2024; Christensen and Pike, 2019), the findings are mixed, with evidence that the deleterious CNS effects of obesity are worsened in *APOE4* carriers in some studies but in *APOE4* non-carriers in others.

One key factor contributing to the disparate observations is biological sex. Sex is known to interact with the *APOE* genotype. There is a significant female bias for many neural effects of *APOE*, ranging from AD risk to depression to changes in white matter volume (Valencia-Olvera et al., 2023; Delano-Wood et al., 2008; Takeuchi et al., 2021). Interestingly, females often show greater *APOE*-related CNS effects of obesity than males (Pandit et al., 2024; Espeland et al., 2021). The consequences of *APOE* on females may be particularly significant at the onset of age-associated changes in the levels of and altered responsiveness to the primary estrogen hormone, 17β-estradiol (17βE2). In women, the perimenopause transition is linked with numerous health implications including increased vulnerability to AD (Pike, 2017) and development of central adiposity and associated cardiometabolic risks (Palacios et al., 2024). Conversely, estrogen-based hormone therapies have been shown to have multi-systemic effects relevant to protection against both AD and systemic metabolic outcomes (Valencia-Olvera et al., 2023; Nerattini et al., 2023; Anagnostis and Goulis, 2019). Certainly, the use of estrogen-based therapies is not without controversy (Koire et al., 2022), but a greater understanding of its risks and benefits is needed, especially in the contexts of *APOE* genotype and obesity.

In the current study, we seek to gain a greater understanding of the relationships among *APOE* genotype, obesity, and estrogen-based treatment in females. To accomplish this, we compared systemic and neural effects of an obesogenic, high-fat diet (HFD) in the presence and absence of treatment with 17βE2 on female *APOE* knock-in mice at an age just prior to the onset of reproductive senescence. Importantly, our study design allowed the testing of the effects of 17βE2 after the establishment of obesity across *APOE* genotypes. Because the number of *APOE4* alleles likely affects many metabolic and neural endpoints but has not been well investigated in human populations and not at all in rodent studies, we also considered possible differences between homo- and heterozygous *APOE4* status by studying mice with *APOE3/3*, *APOE3/4*, and *APOE4/4* genotypes.

## 2 Methods

### 2.1 Animals

**Figure 1:**
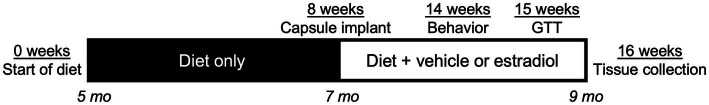
Timeline of experiments. At Week 0 of the study, female *APOE3/3*, *APOE3/4*, and *APOE4/4* knock-in mice at age 5 mo began a 16-week exposure to control or high-fat diet (HFD). At Week 8 (animal age 7 mo), animals were implanted with a Silastic capsule containing vehicle or 17β-estradiol. At Week 14 (6 weeks after capsule implant), animals were behaviorally assessed. One week later, animals were administered an oral glucose tolerance test. Finally, at Week 16 (8 weeks after capsule implant, 16 total weeks of diet, animal age 9 mo), animals were euthanized and tissues were collected for analyses.

A colony of EFAD mice, which have homozygous knock-in of human *APOE* and hemizygous overexpression of AD transgenes from the 5xFAD model (*APOE* ^+/+^, 5xFAD^+/−^) (Youmans et al., 2012), were maintained at vivarium facilities at University of Southern California from breeder mice generously provided by Dr. Mary Jo LaDu (University of Illinois at Chicago). Breeding protocols for EFAD mice yield litters in which ~50% retain *APOE* knock-in but are non-carriers of AD transgenes (*APOE* ^+/+^, 5xFAD^−/−^). Female EFAD non-carriers (*APOE* mice) with *APOE 3/3*, *3/4*, and *4/4* genotypes were studied; *APOE3/4* mice were generated by breeding *APOE3/3* with *APOE4*/*4* mice. Mice were maintained under controlled temperature, on a 12:12-h light/dark schedule (with lights on at 0600), and had *ad libitum* access to food and water. At 5 months of age, female *APOE3/3*, *APOE3/4*, and *APOE4/4* mice were randomly assigned to either control (10% calories from fat and 7% from sugar; catalog #D12450J, Research Diets, Inc., New Brunswick, NJ, United States) or an ingredient-matched high-fat diet (HFD; 60% calories from fat and 7% from sugar; catalog #D12492i, Research Diets, Inc.) (*n* = 22/group). After 8 weeks of diet, animals were subcutaneously implanted between the shoulder blades with either a vehicle (cholesterol) or 17βE2-filled Silastic capsule (25% w/w 17βE2, 75% w/w cholesterol; 1.47 mm ID × 1.96 mm OD; Dow Corning, Midland, MI; *n* = 11/group) to administer constant release of hormone (Christensen et al., 2020). Animals were euthanized 8 weeks later, resulting in 16 total weeks of diet exposure. The experimental procedure is outlined in Figure 1. All procedures were conducted under a protocol (#20648) approved by the USC Institution for Animal Care and Use Committee and under the supervision of USC veterinarians.

### 2.2 Glucose tolerance test

A glucose tolerance test was performed at Week 15 (7 weeks after the start of hormone treatment). Animals were fasted overnight (~16 h) and orally gavaged with 2 g/kg D-glucose. Blood glucose levels were measured at 0, 15, 30, 60, and 120 min following glucose administration. Five microliters of blood were collected on a glucose test strip and assayed using a Precision Xtra Glucose Monitor (Abbott Laboratories, Abbott Park, IL, United States).

### 2.3 Tissue collection

At the end of the 16-week treatment period, mice were euthanized following overnight fasting, after which the brain was rapidly removed. One hemibrain was fixed for 72 h in 4% paraformaldehyde for immunohistochemistry, and the other was rapidly dissected and frozen. Blood was collected and kept on ice prior to centrifugation to collect plasma, which was stored in aliquots at −80° until assayed. The retroperitoneal and visceral (which included gonadal and uterine fat) fat pads were dissected, weighed, and frozen.

### 2.4 Immunohistochemistry

Fixed hemibrains were sectioned exhaustively in the horizontal plane at 40 μm using a vibratome (Leica Biosystems). The sections were stored singly in PBS with 0.03% sodium azide at 4°C until immunohistochemistry was performed. Every eighth section (from a total of ~100 per brain) was immunostained with the following primary antibodies: doublecortin (1:2,500; Santa Cruz) as a marker of new neurons, and Iba-1 (1:2,000; Wako) for microglia. In brief, the tissue sections containing the hippocampus were washed three times for 5 min in TBS, followed by a 10-min rinse with an endogenous peroxidase-blocking solution. Next, the sections were rinsed in TBS/0.1% Triton-X before being incubated for 30 min in a blocking solution consisting of TBS/2% bovine serum albumin or normal horse serum. The sections were incubated overnight at 4°C with the primary antibody diluted in a blocking solution. The sections incubated in the primary antibody were washed and then incubated with the appropriate biotinylated secondary antibody (Vector Laboratories) for 1 h and processed for diaminobenzidine visualization using a Vectastain ABC Elite kit (Vector Laboratories). The stained sections were air-dried overnight, dehydrated in a series of graded alcohols, and then coverslipped with Krystalon (EMD Millipore). One brain from the *APOE3/3* HFD vehicle group was damaged during the sectioning process and could not be used for further analysis.

### 2.5 Quantification of immunolabeled cells

Doublecortin (DCX)-immunoreactive cells were counted in four brain sections that contained the dentate gyrus. Positive labeling was defined as cells that were darkly stained across the majority of the soma. The DCX-labeled cells were counted across the entire dentate gyrus. Cell bodies were mostly found in the subgranular zone or granule cell layer. For microglia, the activated phenotype was based on morphological analysis of Iba-1 immunoreactive cells, as previously described (Christensen and Pike, 2018; Christensen and Pike, 2017). The density of Iba-1 immunoreactive cells in the hippocampus was estimated by two-dimensional counts. In brief, an Olympus BX50 microscope equipped with a motorized stage and computer-guided CASTGrid software (Olympus) was used for unbiased sampling. In the four brain sections per animal, the area containing the subiculum and CA1-CA3 subregions of the hippocampus (excluding the dentate gyrus) was sampled at high magnification. Within each field, the cells within a counting frame (3,000 μm^2^) were used for analysis. Microglia were classified as either type 1 (many thin, ramified processes), type 2 (short, thick processes and a rod-shaped cell body), or type 3 (no or few short non-ramified processes or many filapodial processes) cells. Type 2 and 3 cells were considered to exhibit an activated microglia morphological phenotype.

### 2.6 ELISAs

Fasting levels of leptin, adiponectin, and insulin in plasma collected at euthanization were determined using mouse leptin (Millipore, Catalog # EZML-82 K), adiponectin (Millipore, Catalog #EZMADP-60 K), and insulin (Millipore, Catalog #EZRMI) ELISA kits according to the manufacturer’s instructions. Each animal and standard were tested in duplicate. For the insulin ELISA, values from eight animals were significantly above background (>0.1 ng/mL) but slightly below the lowest standard (0.2 ng/mL); these were assigned a value of 0.2 ng/mL. One animal was excluded due to an exceedingly high level (>3 standard deviations above the mean). For the leptin ELISA, two animals were excluded owing to values at or near the background level.

### 2.7 Lipidomics

Shotgun lipidomics of plasma was performed by the UCLA Lipidomics Core. A modified Bligh and Dyer extraction (Hsieh et al., 2020) was carried out on all plasma samples. Prior to biphasic extraction, an internal standard mixture consisting of 70 lipid standards across 17 subclasses was added to each sample (AB Sciex 5040156, Avanti 330827, Avanti 330830, Avanti 330828, Avanti 791642). Following two successive extractions, pooled organic layers were dried down in a Thermo SpeedVac SPD300DDA using ramp setting 4 at 35°C for 45 min with a total run time of 90 min. Lipid samples were resuspended in 1:1 methanol/dichloromethane with 10 mM ammonium acetate and transferred to robovials (Thermo 10800107) for analysis. The samples were analyzed on the Sciex 5500 with a DMS device (Lipidyzer Platform) with an expanded targeted acquisition list consisting of 1,450 lipid species across 17 subclasses. The differential mobility device on Lipidyzer was tuned with EquiSPLASH LIPIDOMIX (Avanti 330731). The instrument methods including settings, tuning protocol, and MRM list were as previously described (Su et al., 2021).

A total of 862 lipid species were originally detected and subjected to lipidomic bioinformatics analyses. After removing lipids not detected across all samples, 515 remained. The dataset was first normalized to the amount of plasma; then, variance stabilizing normalization was applied to the data using “limma” v.3.48.3, as recommended by previous studies (Jauhiainen et al., 2014; Li et al., 2016). Differential analysis was performed using ‘limma’ in R. Lipids with an FDR < 5% were considered statistically significant. Lipid ontology enrichment analysis was performed using the LION web-based ontology enrichment tool with all detected lipids that passed filtering used as the background (Molenaar et al., 2019).

### 2.8 Behavior

At Week 14, animals were behaviorally assessed using (i) spontaneous alternation behavior test in a Y-maze (SAB), (ii) novel object placement (NOP), and (iii) novel object recognition (NOR) (Figure 1). The spontaneous alternation test is dependent upon the hippocampus and other limbic structures (Lalonde, 2002) and assesses spatial memory and attention toward novelty (Hughes, 2004). Animals were allowed to acclimate to the behavior room for 30 min prior to testing. Next, animals were placed in the long arm of a Y-maze facing away from the other arms to start the test. Arm entries (at least two paws placed into an arm) were recorded for 5 min. Animals with fewer than 20 or more than 50 arm entries were excluded from the analysis. Percent spontaneous alternation was calculated as the number of correct triplicates divided by the total number of triplicate arm entries.

NOP and NOR are cognitive measures that assess learning and memory, involving the hippocampal and parahippocampal regions (Antunes and Biala, 2012). Prior to each day of testing, animals were habituated to the room for at least 30 min. Mice were habituated to the apparatus without objects for 5 min a day for 3 days prior to testing. On the test day, two identical objects were placed into the box, and the mice were allowed to explore for 5 min (sampling). After 15 min, the animals were placed again in the chamber with the same objects, but one object was moved 90 degrees in the chamber (NOP). Mice were allowed to explore for 5 min. Fifteen minutes after NOP, the mice are placed in the chamber with one familiar object and one novel object for 5 min (NOR). The time the mice spent exploring the objects was recorded, and the discrimination index was calculated as (time with novel – time with familiar)/total time with objects. Animals were excluded if they did not spend at least 8 s exploring the objects or did not spend at least some time with each object.

### 2.9 Statistics

All data are reported as the mean ± the standard error of the mean as well as values from individual animals where suitable. The data were analyzed using GraphPad Prism version 8. To compare the effects of experimental treatments across *APOE* genotypes, most data were statistically analyzed using three-way ANOVA followed by Tukey’s *post-hoc* tests when appropriate. Three-way repeated measure ANOVA followed by Tukey’s *post-hoc* tests was used to analyze data measured over time (body weight and GTT). These statistical analyses are listed in Supplementary Table S1. For comparison, the effects of experimental treatments were also assessed within individual *APOE* genotypes using two-way ANOVA followed by Tukey’s *post-hoc* tests when appropriate (Supplementary Table S2).

## 3 Results

### 3.1 17β-estradiol reduces diet-induced increases in body weight and adiposity across APOE genotypes

To investigate how vulnerability to obesity in female mice is affected by increasing *APOE4* allelic dosage, *APOE3/3*, *APOE3/4*, and *APOE4/4* mice were fed either a control or high-fat diet (HFD) beginning at age 5 months (Week 0), which corresponds with peak ovarian cyclicity in virgin mice (Nelson et al., 1982). To further assess estrogen regulation of this relationship, mice were implanted with a delivery capsule containing either vehicle or 17βE2 at age 7 months (Week 8) (Figure 1), the earliest timepoint of age-related irregular cycling and decreased fecundity in mice (Finch, 2014).

**Figure 2:**
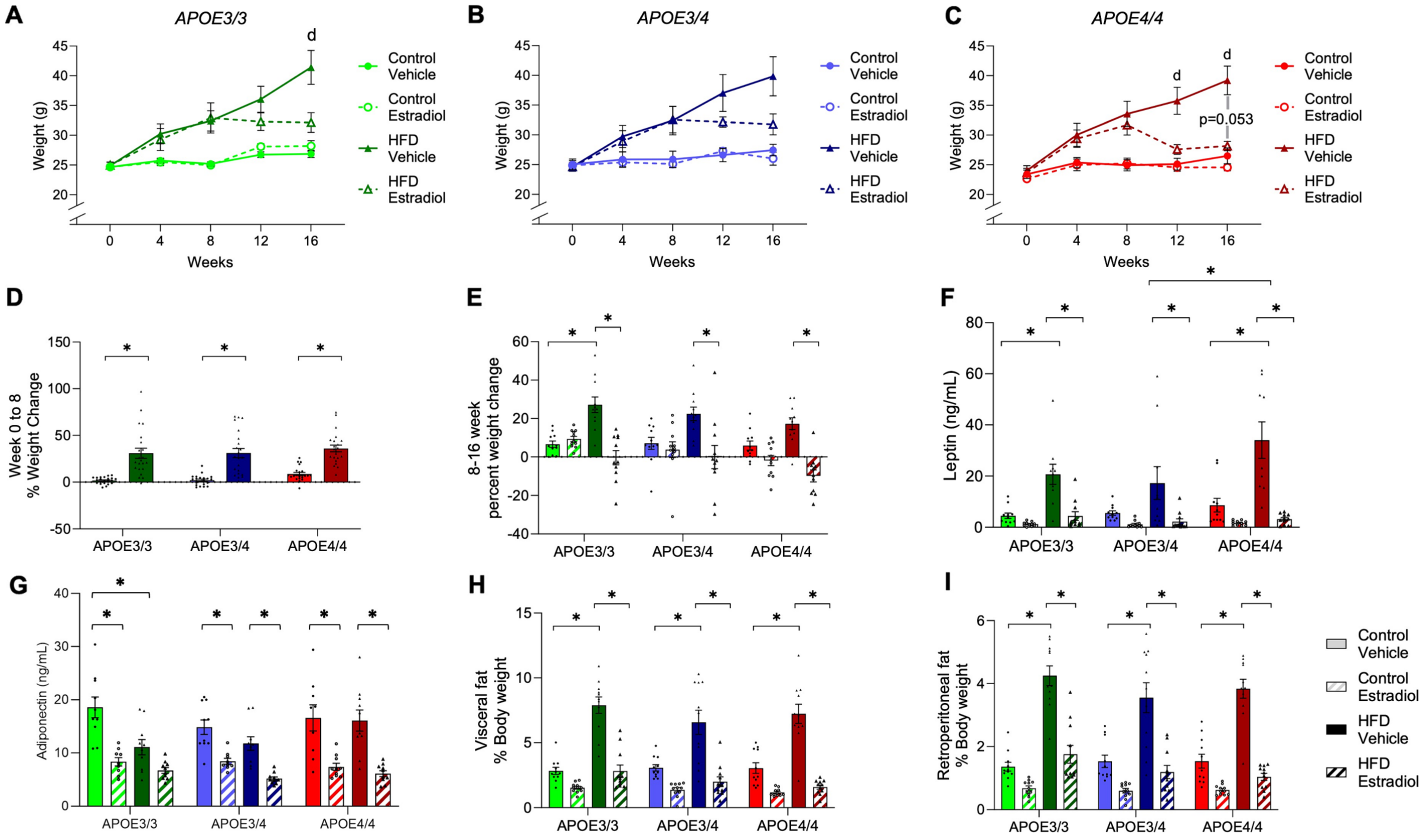
Effects of *APOE*, HFD, and estradiol on body weight. **(A–C)** Body weight was measured weekly. **(D)** The percent weight change was measured for Weeks 0–8 of the experiment, prior to capsule implant. **(E)** The percent weight gain was measured for Weeks 8–16 of the experiment, after capsule implant. **(F)** Plasma levels of leptin and **(G)** adiponectin were measured at the end of the experiment. **(H)** Visceral and **(I)** retroperitoneal fat masses were determined at Week 16 and are represented as the percentage of body weight. The lines and bars colored green are used for *APOE3/3* mice, blue for *APOE3/4* mice, and red for *APOE4/4* mice. The lighter shades represent the control diet, and the darker shades represent HFD. The solid lines and bars represent vehicle capsules, and the hatched lines and bars represent 17β-estradiol capsules. All data are represented as the mean ± SEM with values shown for individual animals where appropriate; *n* = 11 per group. Statistical significance with *p* < 0.05 is denoted by asterisks. Brackets indicate significant differences between specified groups. “d” represents significant differences between control vehicle and HFD vehicle groups. In panel C, “*p* = 0.053” identifies the statistical trend between the HFD vehicle and HFD estradiol groups.

During Weeks 0–8, animals in all three genotypes maintained on HFD gained significantly more weight than genotype-matched mice on the control diet (Figures 2A–D; Supplementary Tables S1, S2). During Weeks 8–16 when 17βE2 or vehicle treatments were present, HFD continued to result in further increases in body weight with vehicle treatment (Figures 2A–C,E); however, this increase was only significant relative to the matched control diet group in *APOE3/3* mice (Figure 2E). Treatment with 17βE2 did not significantly affect body weight with control diet but blunted HFD-induced increases in body mass (Figures 2A–C). In *APOE3/3* and *APOE3/4* mice, 17βE2 completely halted HFD-induced weight gain across weeks 8–16 (Figure 2E). This 17βE2 effect was stronger in *APOE4/4* mice, in which there was a non-significant trend for 17βE2 to reduce body weight (*p* = 0.053; Figures 2C,E) such that mice-fed HFD had lowered body mass to levels very similar to the control diet (Figure 2C).

At Week 16, adipose tissues were collected and weighed. HFD increased visceral and retroperitoneal fat pad weights at similar relative levels in mice from all three genotypes (Figures 2H–I). There was a main effect of 17βE2 treatment across diets, but the decrease in fat pad weight induced by 17βE2 reached statistical significance only in the HFD-fed animals (Supplementary Table S1); 17βE2 reduced adiposity in *APOE4/4* mice on the control diet when statistical analyses were performed within genotypes (Supplementary Table S2) rather than across genotypes (Supplementary Table S1). There were no statistically significant differences in adipose-lowering actions of 17βE2 across the *APOE* genotypes. We also measured plasma levels of leptin, a hormone secreted by adipose tissue that regulates feeding behavior and other functions (Friedman, 2019). There was a significant effect of the *APOE* genotype with *APOE4/4* mice having the highest concentrations of leptin (Figure 2F). Treatment with 17βE2 reduced plasma leptin in both diets and all three genotypes, though the effects were significant only in the context of HFD (Figure 2F). Adiponectin is also released by adipose tissue and has been correlated with metabolic disorder and longevity (Nguyen, 2020; Atzmon et al., 2008). There was a main effect of diet on adiponectin levels, with *post-hoc* analyses showing a significant decrease in adiponectin levels due to HFD only in *APOE3/3* mice. In contrast, *APOE3/4* mice showed a non-significant decrease, and no reduction was observed in *APOE4/4* mice (Figure 2G). Estradiol treatment decreased adiponectin levels even further regardless of diet or genotype (Figure 2G).

### 3.2 17β-estradiol improves diet-induced glucose intolerance most strongly in APOE4 carriers

**Figure 3:**
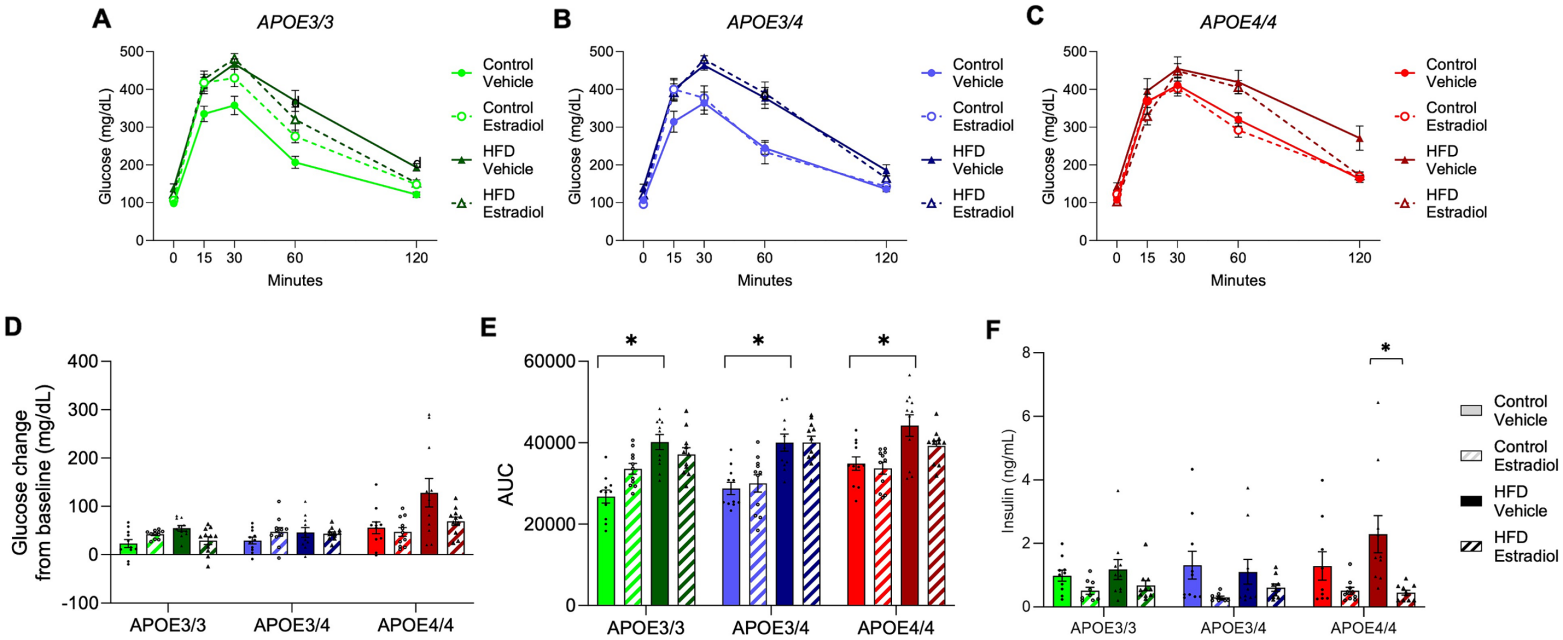
Effects of *APOE*, obesogenic diet, and estradiol on glucose clearance. **(A–C)** An oral glucose tolerance test was administered at Week 15. Blood glucose was measured 15, 30, 60, and 120 min after glucose administration. **(D)** The change in glucose concentration 2 h after glucose administration and **(E)** the area under the curve (AUC) were calculated from the glucose tolerance test. **(F)** Fasting plasma insulin was measured at the end of the experiment. The lines and bars colored green are used for *APOE3/3* mice, blue for *APOE3/4* mice, and red for *APOE4/4* mice. Lighter shades represent the control diet, and darker shades represent HFD. The solid lines and bars represent vehicle capsules, and the hatched lines and bars represent 17β-estradiol capsules. All data are represented as the mean ± SEM with values shown for individual animals where appropriate; *n* = 11 per group. Statistical significance with *p* < 0.05 is denoted by asterisks. Brackets indicate significant differences between specified groups.

To assess glucose metabolism, an oral glucose tolerance test was administered at week 15. HFD significantly impaired glucose clearance in all genotypes with evidence of modest regulation by 17βE2 (Figures 3A–C). Analysis of cumulative glucose clearance over time using area under the curve (AUC) showed a main effect of the *APOE* genotype. For the control diet with vehicle treatment, the lowest AUC was observed in *APOE3/3*, the highest was observed in *APOE4/4*, and intermediate levels were observed in *APOE3/4* (Figure 3E). Notably, there was a significant three-way interaction among diet, *APOE* genotype, and 17βE2 treatment in which 17βE2 improved glucose tolerance specifically within *APOE4* mice-fed HFD (Figure 3E). A similar relationship was observed when comparing the initial vs. final glucose values, which reflect the ability to return glucose levels to baseline levels. By this metric, both HFD-induced impairment and 17βE2 protection were observed only in *APOE4* mice-fed HFD (Figure 3D). Fasting insulin was significantly decreased by estradiol in all genotypes (Figure 3F).

### 3.3 Effects of diet and 17β-estradiol on plasma lipids are strongest in APOE4 carriers

**Figure 4:**
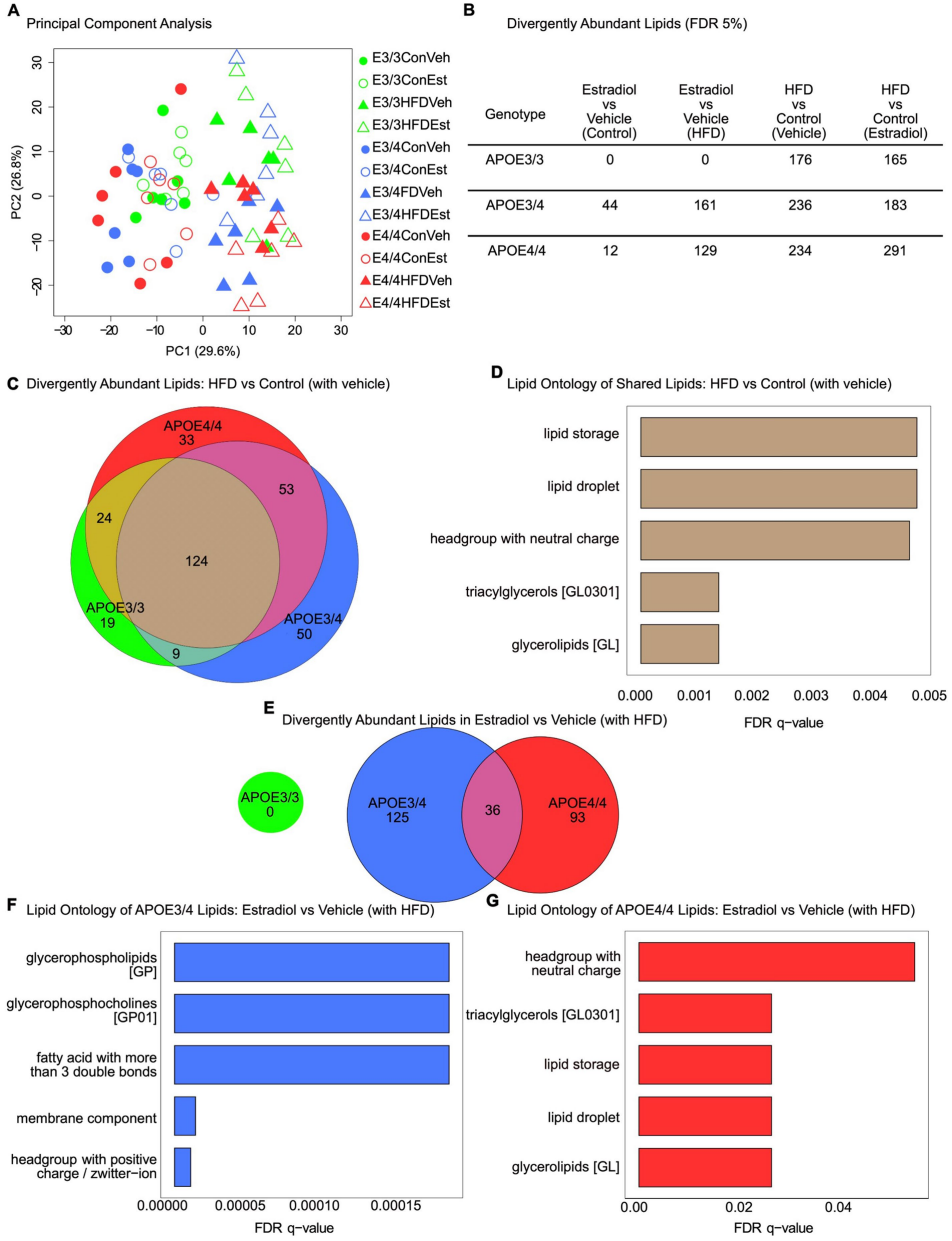
Effects of *APOE* genotype, HFD, and estradiol on plasma lipid profiles. Plasma samples collected at Week 16 were assessed by shotgun lipidomics. **(A)** Principal component analysis of lipidomes for plasma from all 12 groups. **(B)** Table of differentially abundant lipids (FDR 5%) across all relevant comparisons. **(C)** Venn diagram of differentially abundant lipids in the HFD vs. control (with vehicle) comparison. **(D)** LION lipid ontology analysis top enriched features in the shared lipids of the HFD vs. control (with vehicle) comparison. **(E)** Venn diagram of divergently abundant lipids in the estradiol vs. vehicle (with HFD) comparison. **(F)** LION lipid ontology analysis top enriched features in *APOE3/4* estradiol vs. vehicle (with HFD) comparison. **(G)** LION lipid ontology analysis top enriched features in *APOE4/4* estradiol vs. vehicle (with HFD) comparison. For all panels, green is used for *APOE3/3* mice, blue for *APOE3/4* mice, and red for *APOE4/4* mice. For **(A)**, circles represent the control diet, triangles represent HFD, shaded shapes represent vehicle capsules, and unshaded shapes represent 17β-estradiol capsules; *n* = 6 per group.

Given apoE’s key role as a regulator of the lipidome, we performed shotgun lipidomics on plasma from animals of all groups. Principal component analysis showed a clear separation of samples by diet, regardless of 17βE2 treatment or *APOE* genotype (Figure 4A). Examining changes in overall lipid classes, we found that multiple classes were affected by diet, 17βE2 treatment, genotype, and interactions among these factors (Supplementary Figure S1). Fatty acid (FA), ceramide (Cer), phosphatidylcholine (PC), phosphatidylinositol (PI), and phosphatidylethanolamine (PE) were significantly altered based on diet and *APOE* genotype, with Cer, PC, and PI also having significant interactions between diet, genotype, and 17βE2 treatment (Supplementary Figures S1A–E). In addition, there were significant main effects of *APOE* genotype and 17βE2 treatment on hexosyl ceramide (HexCer) levels, with 17βE2 generally increasing HexCer levels, especially when combined with HFD in the *APOE3/4* mice (Supplementary Figure 1F). Abundances of triglycerides (TG) and phosphatidylglycerol (PG) showed main effects of *APOE* genotype, with TGs also having a significant interaction between genotype, diet, and 17βE2 treatment (Supplementary Figures 1G–H). Treatment with HFD had significant main effects on cholesterol ester (CE), diacylglycerol (DG), phosphatidic acid (PA), lyso-phosphatidylethanolamine (LPE), and lyso-phosphatidylcholine (LPC) (Supplementary Figures 1I–M). DGs were also significantly affected by 17βE2, with 17βE2 increasing DG levels. Both DG and PA had an interaction between genotype and diet, with DGs decreasing with HFD only in the *APOE3/4* and *APOE4/4* groups, and PA decreasing with HFD only in the *APOE4/4* (Supplementary Figures 1J–K). There was an interaction between genotype and 17βE2, as well as among genotype, 17βE2, and diet, in LPE levels. Specifically, LPEs decreased only in *APOE3/4* mice on HFD. Combining 17βE2 with HFD increased LPE levels in the *APOE3/4* mice but decreased LPE in *APOE4/4* (Supplementary Figure S1). There were no changes in plasma sphingomyelin levels across any groups (Supplementary Figures S1N).

To better understand the specific effects of experimental factors on the lipidome, we identified lipids differentially abundant by condition within each *APOE* genotype at a false discovery rate (FDR) < 5% (Figure 4B). Notably, 17βE2 treatment resulted in significant differences in plasma lipid species abundance in both *APOE3/4* and *APOE4/4* mice but not in *APOE3/3* mice (Figure 4B). HFD significantly impacted the lipidome in all three *APOE* genotypes, regardless of 17βE2 treatment. We compared the lipids significantly changed by HFD and found 124 shared lipids changed across genotypes (Figure 4C). Enrichment analysis using LION revealed that these shared lipids are primarily involved with lipid storage and lipid droplets and contain several triacylglycerols and glycerolipids (Figure 4D, FDR < 5%). The 17βE2 treatment had no effect on the lipidome in either the control or HFD *APOE3/3* groups but had an additive effect in the *APOE3/4* and *APOE4/4* groups. Specifically, HFD combined with 17βE2 resulted in increased changes in lipid amounts compared to HFD and vehicle treatment. We examined the overlap between these lipids changed in the *APOE3/4* and *APOE4/4* groups and found little commonalities (Figure 4E). The LION enrichment analysis showed that the lipids changed by 17βE2 in the presence of HFD in the *APOE3/4* group are enriched for glycerophospholipids, glycerophosphocholines, membrane component, and others (Figure 4F). In the *APOE4/4* group, lipids changed by 17βE2 in the presence of HFD are enriched for headgroup with neutral charge, triacylglycerols, lipid storage, and lipid droplet (Figure 4G), which is similar to the lipids changed by HFD alone (Figure 4D).

### 3.4 Effects of diet and 17β-estradiol on behavioral performance across APOE genotypes

**Figure 5:**
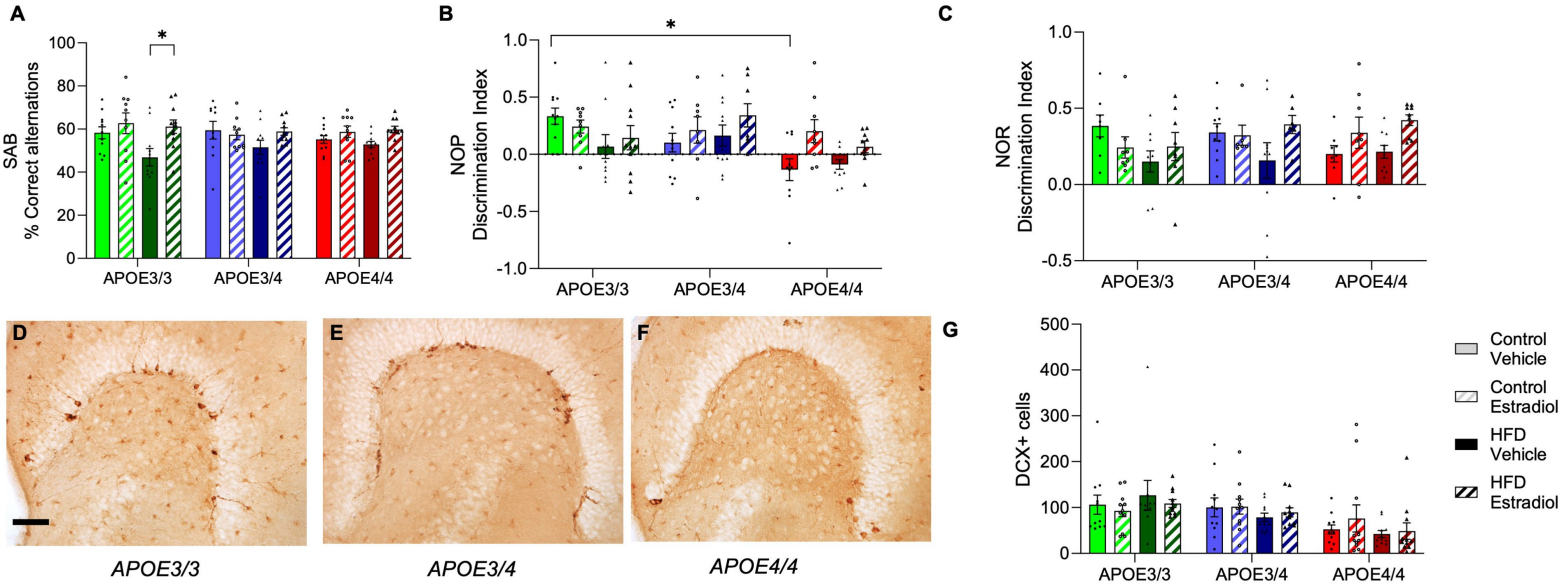
Effects of *APOE* genotype, HFD, and estradiol on behavior and neurogenesis. All behavior analyses were performed at Week 14. **(A)** Spontaneous alternation behavior (SAB) performance is shown as percent correct alternations. The results of **(B)** novel object placement (NOP) and **(C)** novel object recognition (NOR) are presented as the discrimination index. Representative images of doublecortin (DCX) immunostaining in the dentate gyrus from **(D)** *APOE3/3* mice, **(E)** *APOE3/4* mice, and **(F)** *APOE4/4* mice. **(G)** Density of DCX-immunopositive cells was counted in the dentate gyrus. The scale bar is 100 μm in length. Bars colored green are used for *APOE3/3* mice, blue for *APOE3/4* mice, and red for *APOE4/4* mice. Lighter shades represent the control diet, and darker shades represent HFD. The solid bars represent vehicle capsules, and the hatched bars represent 17β-estradiol capsules. All data are represented as the mean ± SEM with values shown for individual animals; *n* = 6–11 per group. Statistical significance with *p* < 0.05 is denoted by asterisks. Brackets indicate significant differences between specified groups.

At Week 14, all mice were examined behaviorally using the SAB, NOP, and NOR tasks, all of which involve aspects of hippocampal function (Lalonde, 2002; Hughes, 2004; Antunes and Biala, 2012). In SAB, only *APOE3/3* mice showed significant behavioral effects of diet and 17βE2 treatment. Specifically, there was a significant diet X hormone interaction in which 17βE2 improved SAB performance with HFD exposure specifically in *APOE3/3* mice, and the genotype was also associated with the largest HFD-associated impairment (Figure 5A).

For the NOP task, there were significant main effects of both *APOE* genotype and hormone treatment (Figure 5B). On the control diet, *APOE4/4* mice performed significantly poorer than *APOE3/3* mice with intermediate deficits observed in *APOE3/4* mice. Treatment with 17βE2 significantly improved NOP performance with the strongest improvements in *APOE4/4* mice and the weakest benefits in *APOE3/3* mice (Figure 5B). Diet was not associated with significant main or interactive effects, but as in SAB, *APOE3/3* mice trended toward showing the most HFD-associated impairment. Unlike NOP, NOR was not associated with a significant main effect of the *APOE* genotype. There was a significant interaction between diet and hormone treatment such that 17βE2 increased performance in the presence of HFD, an effect that trended toward stronger effects in *APOE4* carriers (Figure 5C).

Because neurogenesis in the hippocampal subgranular zone is positively associated with cognitive behaviors (Aimone et al., 2014; Villeda et al., 2011) and 17βE2 (Sahab-Negah et al., 2020) but negatively impacted by *APOE4* (Li et al., 2009) and HFD (Robison et al., 2020; Lindqvist et al., 2006), we determined whether differences in neurogenesis across groups may predict behavioral performances. Numbers of neural stem cells committed to neuronal differentiation were quantified by counts of DCX-positive cells in the dentate gyrus. There was a significant effect of the *APOE* genotype with *APOE4/4* mice having fewer DCX-positive cells than *APOE3/3* and *APOE3/4* mice (Figures 5D–G). There were no significant main or interactive effects of diet or hormone treatment.

### 3.5 Microglia are differentially affected by diet and 17β-estradiol across APOE genotypes

**Figure 6:**
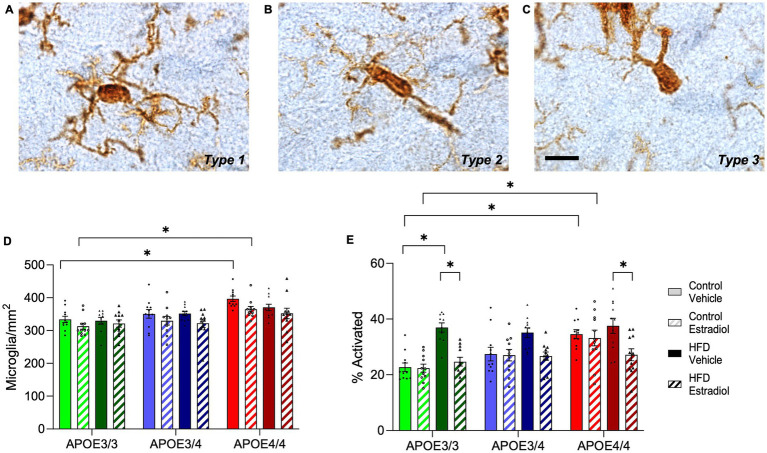
Effects of *APOE* genotype, HFD, and estradiol on microglia number and activation phenotype. Cells immunolabeled with Iba-1 were morphologically characterized as microglia with **(A)** type1 (homeostatic), **(B)** type 2 (activated), or **(C)** type 3 (activated) phenotypes. **(D)** The cell density and **(E)** percent activation of Iba-1 labeled microglia (relative abundance of types 2 and 3) were determined in the hippocampus. The scale bar is 10 μm in length. Bars colored green are used for *APOE3/3* mice, blue for *APOE3/4* mice, and red for *APOE4/4* mice. Lighter shades represent control diet, and darker shades represent HFD. The solid bars represent vehicle capsules, and the hatched bars represent 17β-estradiol capsules. All data are represented as the mean ± SEM with values shown for individual animals; *n* = 10–11 per group. Statistical significance with *p* < 0.05 is denoted by asterisks. Brackets indicate significant differences between specified groups.

Because microglial phenotypes are implicated in cognitive performance (McKee et al., 2023; Blank and Prinz, 2013) and are regulated by HFD (Alexaki, 2021), *APOE4* genotype (Lee et al., 2023; Ferrari-Souza et al., 2023), and 17βE2 (Bruce-Keller et al., 2000; Mor et al., 1999), we next measured the numbers of total and morphologically activated microglia in the hippocampus. Interestingly, the number of microglia differed by both *APOE* genotype and estradiol treatment, with *APOE4/4* increasing and 17βE2 treatment decreasing microglia density (Figure 6D). *APOE3/4* mice showed an intermediate number of microglia and were not significantly different from *APOE3/3* or *APOE4/4* mice. Activation phenotype of microglia was measured by morphology as previously described (Moser and Pike, 2017; Christensen and Pike, 2019; Christensen and Pike, 2017; Ekdahl, 2012). Type 1 microglia (Figure 6A) have many long, thin ramified processes and are considered functionally homeostatic, whereas Types 2 (Figure 6B) and 3 (Figure 6C) have shorter and fewer processes, with Type 3 being associated with activated phenotypes. The microglial phenotype was significantly influenced by *APOE* genotype, diet, and hormones, with interactive effects of *APOE* and diet as well as diet and hormone (Figure 6E). Specifically, in mice maintained on the control diet, *APOE* genotype was significantly associated with microglial phenotypes with activation increasing in accordance with *APOE4* allele dosage: *APOE3/3 < APOE3/4 < APOE4/4*. 17βE2 did not affect microglial activation under the control diet. HFD was associated with increased activated phenotypes, although the increase was only significantly different from the control diet in *APOE3/3* mice. In HFD-fed animals, 17βE2 treatment decreased activation, with a significant effect observed in both *APOE3/3* and *APOE4/4* mice, although it was most robust in *APOE3/3* mice (Figure 6E).

## 4 Discussion

In this study, we investigated the effects of the *APOE4* genotype on systemic metabolic and CNS effects related to obesity in female mice, as well as the relative efficacy of treatment with the estrogen 17βE2. The design was planned to test the established protective effects of 17βE2 in the context of existing obesity and at an age just prior to the onset of reproductive senescence. The results suggest that for metabolic measures, 17βE2 is most effective in *APOE4* homozygotes. Specifically, 17βE2 protected against HFD-induced increases in body weight, plasma leptin, and glucose intolerance most strongly in *APOE4/4* mice. Conversely, 17βE2 was most effective in ameliorating the adverse neural effects of HFD in *APOE3/3* mice, with significant improvements seen in both the SAB behavioral task and levels of microglial activation. However, the behavioral benefits of 17βE2 were task-dependent, with carriers of *APOE4* but not *APOE3/3* showing evidence of improved performance of the NOP and NOR tasks.

The impacts of *APOE* genotype on responses to obesity have been mixed in prior studies. In humans, evidence regarding the relationship between obesity and dementia or other neural risks is conflicting: some studies suggest these risks are greater in *APOE4* carriers (Mole et al., 2020; Osiecka et al., 2023), while others indicate a more robust effect in *APOE4* non-carriers (Coad et al., 2022; Shinohara et al., 2023; Zhao et al., 2023). The findings from rodent data are also contradictory, with evidence that either *APOE3* (Jones et al., 2021; Pandit et al., 2024) or *APOE4* (Johnson et al., 2017; Arbones-Mainar et al., 2008) is associated with greater obesity-induced impairments. Sex may be an important variable in this relationship (Moser and Pike, 2016; Mattar et al., 2022), a possibility that aligns with our prior observations in the EFAD mouse model (Moser and Pike, 2017; Christensen and Pike, 2019). In this study of female mice, obesity-induced metabolic effects were generally somewhat stronger with *APOE4/4*, whereas the neural effects tended to be more pronounced with *APOE3/3*. One limitation of our assessment of glucose metabolism is that we assessed glucose but not insulin tolerance, which would have provided a more complete understanding of metabolic responses to obesity. In terms of plasma lipids, HFD induced robust changes in lipid abundances across all groups. In agreement with the metabolic findings, lipidomics showed that *APOE4* carriers had greater obesity-induced changes. One limitation is that lipidomic analyses did not assess potential changes in the brain. Collectively, these findings reinforce the position that the *APOE4* genotype yields enhanced vulnerability to obesity.

Perhaps most interesting were the variable effects of *APOE4* heterozygosity across different measures. As might be expected, *APOE3/4* mice showed intermediate values relative to *APOE3/3* and *APOE4/4* mice on some measures, for example on levels of newborn neurons and microglial density and activation. However, on metabolic measures, *APOE3/4* mice were generally more similar to *APOE3/3* than *APOE4/4* mice. For example, like *APOE3/3* mice, *APOE3/4* mice trended toward reduced adiponectin when challenged by HFD, whereas *APOE4/4* showed no effect. In lipidomic analyses, both *APOE3/4* and *APOE4/4* mice showed more robust HFD-induced changes than *APOE3/3*, yet the pattern of alterations often differed between the *APOE4* genotypes suggesting qualitative differences rather than just gene dosage effects. On the SAB task of attention and working memory, *APOE3/4* mirrored *APOE4/4* mice showing negligible effects of both HFD and 17βE2, whereas *APOE3/3* showed an HFD-induced deficit that was rescued by 17βE2. On the NOP and NOR behavioral tests, *APOE3/4* and *APOE3/3* mice showed very similar performance levels under control diet conditions with *APOE4/4* strongly trending toward comparatively impaired performance. Yet the significant diet X hormone interaction on NOP was driven by 17βE2-mediated improvements against HFD in *APOE3/4* and *APOE4/4* mice, with *APOE3/3* mice showing minimal increases.

*APOE4* allele number has significant effects on a range of outcomes in humans, though typically not in a linearly graded manner. For AD risk, the effects of *APOE4/4* are several-fold higher than *APOE3/4* (Reiman et al., 2020). Indeed, recent analyses have revealed near universal expression of AD neuropathology in *APOE4* homozygotes by middle age (Fortea et al., 2024). In non-demented persons, *APOE4* homozygosity significantly accelerates cognitive decline; however, the effects of *APOE4* heterozygosity are comparatively modest (Gharbi-Meliani et al., 2021; Rawle et al., 2018; Caselli et al., 2011). *APOE4* zygosity also impacts neuroimaging and plasma biomarkers of AD in the absence of dementia, with *APOE3/4* carriers generally aligning more closely with *APOE3/3* than *APOE4/4* carriers (Tato-Fernandez et al., 2024). *APOE4* zygosity also appears to impact the positive association between metabolic risk factors and a neuroimaging marker of neuroinflammation as *APOE3/4* carriers are reported to differ from both *APOE3/3* and *APOE4/4* carriers (Ekblad et al., 2023). Notably, despite the significant differences with one vs. two copies of *APOE4*, most human studies of populations do not discriminate but rather pool homo- and heterozygous *APOE4* genotypes into a combined group of *APOE4* carriers.

Studies of rodents with knock-in of human *APOE* alleles are an excellent strategy to address existing knowledge gaps in the field. However, the issue of *APOE4* homo- vs. heterozygosity is not well addressed in the preclinical literature. Few rodent studies have included *APOE3/4* mice, with most comparing *APOE3/3* vs. *APOE4/4* mice. Among those that have considered *APOE4* zygosity, there is not a consistent trend. One study of aged male and female *APOE* knock-in mice reported limited evidence of behavioral differences across *APOE4* zygosity, in part due to high variability within groups (McLean et al., 2022). A recent targeted brain lipidome analysis in middle-aged male *APOE* knock-in mice reported differences across *APOE3/3*, *APOE3/4*, and *APOE4/4* genotypes with *APOE3/4* often positioned between the two homozygous groups (Miranda et al., 2022). In parallel to some of our observations, a cerebrocortical transcriptomic analysis found unique profiles in *APOE3/4* mice (Foley et al., 2022). In the EFAD model that includes Alzheimer’s transgenes with *APOE* knock-in, females at age 6 months were ovariectomized and then studied with acute 17βE2 or vehicle treatments (Taxier et al., 2022). Improved memory on novel recognition tasks was observed in *APOE3/3* and *APOE3/4* but not *APOE4/4* mice, suggesting an absence of 17βE2 neural benefits with *APOE4* homozygosity. These results differ from our findings in the NOP and NOR behavioral tasks, which may reflect several factors including acute vs. chronic 17βE2 treatment and the presence vs. absence of underlying AD-related pathology. Interestingly, the findings of Taxier et al. align with our findings regarding the complex effects of *APOE4* zygosity. They reported *APOE3/4* outcomes varied between *APOE3/3*-like, intermediate, and *APOE4/4*-like values across different metrics of spine density (Taxier et al., 2022). Collectively, our findings add to a limited, but much needed, experimental literature aimed at understanding the differing effects of 0, 1, and 2 copies of *APOE4*.

Another interesting finding from our study concerned the effects of *APOE* genotype on microglia. We made the unexpected finding that, even without outside stressors, *APOE4/4* was associated with a greater cell density of Iba-1 immunolabeled microglia and a higher proportion exhibiting a morphologically activated phenotype. To our knowledge, this finding has not been reported previously in males or females. *APOE3/4* mice appeared to be intermediate and were not significantly different than mice with either homozygous genotype. These observations are consistent with findings from several groups indicating that, relative to *APOE3/3*, *APOE4/4* microglia show a variety of transcriptomic and functional differences linked with poor neural outcomes (Liu et al., 2023; Lee et al., 2023; Machlovi et al., 2022; Stephen et al., 2019). Furthermore, in response to stressors ranging from demyelination (Wang et al., 2022) to systemic immune challenge (Zhu et al., 2012) and AD-related pathology (Rodriguez et al., 2014; Liu et al., 2017), the *APOE* genotype strongly impacts indices of microglia activation.

Importantly, we observed that treatment with 17βE2 was associated with reductions in both microglial numbers and levels of microglial activation regardless of *APOE* genotype. This finding is consistent with prior observations that estrogen treatments decrease inflammatory tone in the brain (Wu et al., 2016; Loiola et al., 2019). Given the noted variability in the effects of *APOE* genotype on numerous outcomes, it is perhaps not surprising that *APOE*-associated modulation on estrogen actions differs across studies. In experimental studies, there are reports that the beneficial effects of 17βE2 are alternatively better (Yun et al., 2007) or poorer (Taxier et al., 2022; Brown et al., 2008; Nathan et al., 2004) in the context of the *APOE4* genotype. Furthermore, there is evidence that 17βE2 treatment increases β-amyloid deposition in *APOE4* mice but improves outcomes in *APOE2* and *APOE3* mice (Kunzler et al., 2014). The current study finds that the relationship between 17βE2 efficacy and *APOE* genotype depends upon the outcome measure. For HFD-induced increases in body weight, plasma leptin, and glucose intolerance, 17βE2 was more beneficial in *APOE4/4* mice. Conversely, *APOE3/3* mice showed the most 17βE2-induced improvement in microglial measures and the SAB behavioral task. In lipidomic analyses, 17βE2 treatment had much greater effects in *APOE4* carriers, with *APOE3/4* mice showing profiles distinct from both *APOE3/3* mice and *APOE4/4* mice. Human studies on the efficacy of estrogen-based therapies are similarly mixed (Moser and Pike, 2016) with greater CNS benefits reported in *APOE4* non-carriers (Yaffe et al., 2000; Burkhardt et al., 2004) or *APOE4* carriers (Saleh et al., 2023; de Lange et al., 2020) across different studies and endpoints. Our study demonstrates that the impact of *APOE4* carrier status depends upon both zygosity and the specific measure. We suggest that integrating the disparities in the human literature requires not only an acknowledgment of the complexities of the interactions between *APOE* genotype and 17βE2 on numerous cell types and systems throughout the body but also a consideration of *APOE4* zygosity. While *APOE4* is generally classified as deleterious and 17βE2 as beneficial, these are overly simplistic descriptions for factors known to exert pleiotropic actions (Patel et al., 2018; Martinez-Martinez et al., 2020).

In conclusion, this study suggests that *APOE* genotype, obesity, and estrogen signaling interact to affect cognitive, metabolic, and lipid outcomes. *APOE4/4* carriers were more responsive to 17βE2 than *APOE3/3* mice on metabolic measures after HFD, whereas *APOE3/3* mice were more responsive to 17βE2 on CNS outcomes. Importantly, heterozygous *APOE3/4* mice showed responses that varied between *APOE3/3*-like and *APOE4/4*-like to a unique *APOE3/4* profile depending upon the outcome measure and the presence and absence of HFD and 17βE2. Given the relative rarity of *APOE4* homozygosity in human populations, these results emphasize the limitation of relying on *APOE4/4* mice as an experimental model. These data add to the limited but much-needed literature on understanding the effects of *APOE4* allele dosage and its interactions with established modulators of systemic and CNS impairment.

## Data Availability

The raw data supporting the conclusions of this article will be made available by the authors, without undue reservation.
