# Supplementary material for: Effects of obesogenic diet and 17β-estradiol in female mice with APOE 3/3, 3/4, and 4/4 genotypes
Source: Front Aging Neurosci. 2024 Sep 13;16:1415072. doi: 10.3389/fnagi.2024.1415072 (PMC11427389; doi:10.3389/fnagi.2024.1415072)
Supplement: Supplementary file 1 [file Table_1.DOCX]

***Supplementary Material***

**Effects of obesogenic diet and 17β-estradiol in female mice with *APOE* 3/3, 3/4 and 4/4 genotypes**

Amy Christensen, Cassandra J. McGill, Wenjie Qian, and Christian J. Pike

**Table 1.** Summary of statistical analyses with comparisons across *APOE* genotypes.

| **Figure** | **Measure**  **Used** | **Main effects and interactions** | **Selected *post hoc* comparisons** |
| --- | --- | --- | --- |
| 2A | 3-way RM ANOVA | Time: F(2.57, 103) = 48.3, p<0.0001  Diet: F(1, 40) = 24.2, p<0.0001  Time X Diet: F(4, 160) = 18.3, p<0.0001  Time X Hormone: F(4, 160) = 4.24, p<0.01  Time X Diet X Hormone: F(4, 160) = 8.40, p<0.0001 | 16:Control Vehicle vs. 16:HFD Vehicle; p<0.05  16:Control Estradiol vs. 16:HFD Vehicle; p<0.05 |
| 2B | 3-way RM ANOVA | Time: F(2.37, 94.9) = 34.4, p<0.0001  Diet: F(1, 40) = 15.1, p<0.001  Time X Diet: F(4, 160) = 17.7, p<0.0001  Time X Hormone: F(4, 160) = 4.71, p<0.01  Time X Diet X Hormone: F(4, 160) = 3.86, p<0.01 |  |
| 2C | 3-way RM ANOVA | Time: F(2.86, 114) = 49.8, p<0.0001  Diet: F(1, 40) = 23.1, p<0.0001  Hormone: F(1, 40) = 4.78, p<0.05  Time X Diet: F(4, 160) = 19.1, p<0.0001  Time X Hormone: F(4, 160) = 16.1, p<0.0001  Time X Diet X Hormone: F(4, 160) = 10.2, p<0.0001 | 16:Control Vehicle vs. 16:HFD Vehicle; p<0.05  16:HFD Vehicle vs. 16:HFD Estradiol; p=0.053 |
| 2D | 2-way ANOVA | Diet: F(1, 126) = 103, p<0.0001 | APOE3/3:Control vs. APOE3/3:HFD; p<0.0001  APOE3/4:Control vs. APOE3/4:HFD; p<0.0001  APOE4/4:Control vs. APOE4/4:HFD; p<0.0001 |
| 2E | 3-way ANOVA | APOE: F(2, 120) = 5.49, p<0.01  Diet: F(1, 120) = 4.72, p<0.05  Hormone: F(1, 120) = 52.3, p<0.0001  Diet X Hormone: F(1, 120) = 33.8, p<0.0001 | APOE3/3:Control Vehicle vs. APOE3/3:HFD Vehicle; p<0.01  APOE3/3:HFD Vehicle vs. APOE3/3:HFD Estradiol; p<0.0001  APOE3/4:Control Vehicle vs. APOE3/4:HFD Vehicle; p= 0.08  APOE3/4:HFD Vehicle vs. APOE3/4:HFD Estradiol; p<0.001  APOE4/4:Control Estradiol vs. APOE4/4:HFD Vehicle; p<0.01  APOE4/4:HFD Vehicle vs. APOE4/4:HFD Estradiol; p<0.0001 |
| 2F | 3-way ANOVA | APOE: F(2, 116) = 3.71; p<0.05  Diet: F(1, 116) = 34.6; p<0.0001  Hormone: F(1, 116) = 58.9; p<0.0001  APOE X Hormone: (2, 116) = 3.33; p<0.05  Diet X Hormone: (1, 116) = 22.6; p<0.0001 | APOE3/3:Control Vehicle vs. APOE3/3:HFD Vehicle; p<0.01  APOE3/3:HFD Vehicle vs. APOE3/3:HFD Estradiol; p<0.01  APOE3/4:HFD Vehicle vs. APOE3/4:HFD Estradiol; p<0.05  APOE3/4:HFD Vehicle vs. APOE4:HFD Vehicle; p<0.01  APOE4/4:Control Vehicle vs. APOE4/4:HFD Vehicle; p<0.0001  APOE4/4:HFD Vehicle vs. APOE4/4:HFD Estradiol; p<0.0001 |
| 2G | 3-way ANOVA | Diet: F(1, 105) = 14.1; p<0.001  Hormone: F(1, 105) = 104.2; p<0.0001 | APOE3/3 Control Vehicle vs. APOE3/3 Control Estradiol; p<0.0001  APOE3/3 Control Vehicle vs. APOE3/3 HFD Vehicle; p<0.01  APOE3/4 Control Vehicle vs. APOE3/4 Control Estradiol; p<0.05  APOE3/4 HFD Vehicle vs. APOE3/4 HFD Estradiol; p<0.05  APOE4/4 Control Vehicle vs. APOE4/4 Control Estradiol; p<0.001  APOE4/4 HFD Vehicle vs. APOE4/4 HFD Estradiol; p<0.0001 |
| 2H | 3-way ANOVA | Diet: F(1, 120) = 89.4, p<0.0001  Hormone: F(1, 120) = 160, p<0.0001  Diet X Hormone: F(1, 120) = 43.0, p<0.0001 | APOE3/3:Control Vehicle vs. APOE3/3:HFD Vehicle; p<0.0001  APOE3/3:HFD Vehicle vs. APOE3/3:HFD Estradiol; p<0.0001  APOE3/4:Control Vehicle vs. APOE3/4:HFD Vehicle; p<0.0001  APOE3/4:HFD Vehicle vs. APOE3/4:HFD Estradiol; p<0.0001  APOE4/4:Control Vehicle vs. APOE4/4:HFD Vehicle; p<0.0001  APOE4/4:HFD Vehicle vs. APOE4/4:HFD Estradiol; p<0.0001 |
| 2I | 3-way ANOVA | Diet: F(1, 120) = 130, p<0.0001  Hormone: F(1, 120) = 155, p<0.0001  APOE X Diet: F(2, 120) = 2.56, p=0.08  Diet X Hormone: F(1, 120) = 39.3, p<0.0001 | APOE3/3:Control Vehicle vs. APOE3/3:HFD Vehicle; p<0.0001  APOE3/3:HFD Vehicle vs. APOE3/3:HFD Estradiol; p<0.0001  APOE3/4:Control Vehicle vs. APOE3/4:HFD Vehicle; p<0.0001  APOE3/4:HFD Vehicle vs. APOE3/4:HFD Estradiol; p<0.0001  APOE4/4:Control Vehicle vs. APOE4/4:HFD Vehicle; p<0.0001  APOE4/4:HFD Vehicle vs. APOE4/4:HFD Estradiol; p<0.0001 |
| 3A | 3-way RM ANOVA | Time: F(2.47, 99.0) = 374, p<0.0001  Diet: F(1, 40) = 25.7, p<0.0001  Time X Diet: F(4, 160) = 4.73, p<0.01  Time X Hormone: F(4, 160) = 3.33, p<0.05  Diet X Hormone: F(4, 40) = 8.34, p<0.01 | 30:Control Vehicle vs. 30:HFD Vehicle; p= 0.08  60:Control Vehicle vs. 60:HFD Vehicle; p<0.01  120:Control Vehicle vs. 120:HFD Vehicle; p<0.01 |
| 3B | 3-way RM ANOVA | Time: F(2.45, 98.1) = 243, p<0.0001  Diet: F(1, 40) = 26.9, p<0.0001  Time X Diet: F(4, 160) = 8.72, p<0.0001 | 60:Control Vehicle vs. 60:HFD Vehicle; p<0.05 |
| 3C | 3-way RM ANOVA | Time: F(2.69, 107) = 221, p<0.0001  Diet: F(1, 40) = 9.14, p<0.01  Hormone: F(1, 40) = 2.99, p= 0.09  Time X Diet: F(4, 160) = 6.30, p<0.0001  Time X Diet X Hormone: F(4, 160) = 2.06, p= 0.09 |  |
| 3D | 3-way ANOVA | APOE: F(1.43, 57.1) = 13.0, p<0.0001  Diet: F(1, 40) = 9.96, p<0.01  APOE X Diet: F(2, 80) = 3.91, p<0.05  APOE X Hormone: F(2, 80) = 3.51, p<0.05  Diet X Hormone: F(1, 40) = 8.33, p<0.01 | APOE3/3:HFD Estradiol vs. APOE4/4:HFD Estradiol; p= 0.052 |
| 3E | 3-way ANOVA | APOE: F(2, 120) = 5.1, p<0.01  Diet: F(1, 120) = 75.45, p<0.0001  Diet X Hormone: F(1, 120) = 6.0, p<0.05 | APOE3/3:Control Vehicle vs. APOE3/3:HFD Vehicle; p<0.0001  APOE3/3:Control Vehicle vs. APOE4/4:Control Vehicle; p=0.06  APOE3/4:Control Vehicle vs. APOE3/4:HFD Vehicle; p<0.001  APOE4/4:Control Vehicle vs. APOE4/4:HFD Vehicle; p<0.05 |
| 3F | 3-way ANOVA | Hormone: F(1, 104) = 23.4; p<0.0001 | APOE4/4 HFD Vehicle vs. APOE4/4 HFD Estradiol; p<0.01 |
| 4A | 3-way ANOVA | Diet: F(1, 115) = 4.07, p<0.05  Hormone: F(1, 115) = 11.2, p<0.01  Diet X Hormone: F(1, 115) = 4.90, p<0.05 | APOE3/3:HFD Vehicle vs. APOE3/3:HFD Estradiol; p<0.05 |
| 4B | 3-way ANOVA | APOE: F(2, 106) = 6.24, p<0.01  Hormone: F(1, 106) = 6.35, p<0.05  APOE X Diet: F(2, 106) = 2.49, p= 0.09 | APOE3/3:Control Vehicle vs. APOE4/4:Control Vehicle; p<0.01 |
| 4C | 3-way ANOVA | Hormone: F(1, 88) = 3.89, p= 0.052  Diet X Hormone: F(1, 88) = 4.55, p<0.05 |  |
| 4G | 3-way ANOVA | APOE: F(2, 119) = 9.59, p<0.001 | APOE3/3:HFD Vehicle vs. APOE4/4:HFD Vehicle; p= 0.059 |
| 5D | 3-way ANOVA | APOE: F(2, 119) = 18.8, p<0.0001  Hormone: F(1, 119) = 11.7, p<0.001 | APOE3/3:Control Vehicle vs. APOE4/4:Control Vehicle; p<0.01  APOE3/3:Control Estradiol vs. APOE4/4:Control Estradiol; p<0.05 |
| 5E | 3-way ANOVA | APOE: F(2, 119) = 10.7, p<0.0001  Diet: F(1, 119) = 9.26, p<0.01  Hormone: F(1, 119) = 23.1, p<0.0001  APOE X Diet: F(2, 119) = 6.01, p<0.01  Diet X Hormone: F(1, 119) = 17.6, p<0.0001 | APOE3/3:Control Vehicle vs. APOE3/3:HFD Vehicle; p<0.001  APOE3/3:Control Vehicle vs. APOE4/4:Control Vehicle; p<0.01  APOE3/3:Control Estradiol vs. APOE4/4:Control Estradiol; p<0.01  APOE3/3:HFD Vehicle vs. APOE3/3:HFD Estradiol; p<0.01  APOE4/4:HFD Vehicle vs. APOE4/4:HFD Estradiol; p<0.05 |
| S1A | 3-way  ANOVA | *APOE*: *F*_(2, 60)_ = 3.85,  *p*<0.05  Diet: F(1,60) = 18.88, *p*<0.0001 |  |
| S1B | 3-way  ANOVA | *APOE*:  *F*_(2, 60)_ = 11.27, *p*<0.0001  Diet:  *F*_(1, 60)_ = 13.39,  *p* <0.01  *APOE* X Hormone: F(2, 60) = 6.94,  *p* <0.01  Diet X Hormone:  *F*_(1, 60)_ = 4.90,  *p* <0.05 | *APOE3/4*:Control Estradiol vs. *APOE3/4*:HFD Vehicle;  *p*<0.05  *APOE3/4:*Control Vehicle vs. *APOE3/4*:HFD Vehicle;  *p*<0.05  *APOE3/4*:HFD Vehicle vs. *APOE3/4*:HFD Estradiol; *p<0.001*  *APOE4/4*:Control Vehicle vs. *APOE4/4*:HFD Estradiol;  *p*<0.05 |
| S1C | 3-way  ANOVA | *APOE*:  *F*_(2, 60)_ = 3.45,  *p* <0.05  Diet:  *F*_(1, 60)_ = 11.38,  *p* <0.01  *APOE* X Hormone: *F*_(2, 60)_ = 5.78,  *p* <0.01  *APOE* X Diet X Hormone:  *F*_(2, 60)_ = 4.55,  *p* <0.05 | *APOE3/4*:HFD Estradiol vs. *APOE4/4*:HFD Estradiol;  *p*<0.05  *APOE4/4*:Control Vehicle vs. *APOE4/4*:HFD Estradiol;  *p*<0.01 |
| S1D | 3-way  ANOVA | *APOE*:  *F*_(2, 60)_ = 4.22,  *p* <0.05  Diet:  *F*_(1, 60)_ = 23.73, *p*<0.0001  *APOE* X Diet X Hormone:  *F*_(2, 60)_ = 5.95,  *p* <0.01 | *APOE3/4*:Control Vehicle vs. *APOE3/4*:HFD Vehicle;  *p*<0.05  *APOE3/4*:Control Estradiol vs. *APOE3/4*:HFD Vehicle;  *p*<0.05  *APOE3/4*:HFD Estradiol vs. *APOE4/4*:HFD Estradiol;  *p*<0.05  *APOE4/4*:Control Estradiol vs. *APOE4/4*:HFD Estradiol;  *p*<0.05 |
| S1E | 3-way  ANOVA | *APOE*:  *F*_(2, 60)_ = 5.37,  *p* <0.01  Diet:  *F*_(1, 60)_ = 17.65, *p*<0.0001 | *APOE4/4*:Control Vehicle vs. *APOE4/4*:HFD Estradiol;  *p*<0.05 |
| S1F | 3-way  ANOVA | *APOE*:  *F*_(2, 60)_ = 6.62, p=0.0025  Hormone:  *F*_(1, 60)_ = 21.13, *p*<0.0001 | *APOE3/4*:Control Vehicle vs. *APOE3/4*:HFD Estradiol;  *p*<0.05  *APOE3/4*:HFD Vehicle vs. *APOE3/4*:HFD Estradiol;  *p*<0.001 |
| S1G | 3-way  ANOVA | *APOE*:  *F*_(2, 60)_ = 3.53,  *p*<0.05  *APOE* X Diet X Hormone:  *F*_(2, 60)_ = 3.94,  *p*<0.05 |  |
| S1H | 3-way  ANOVA | *APOE*:  *F*_(2, 60)_ = 7.00, *p*<0.01 |  |
| S1I | 3-way  ANOVA | Diet:  *F*_(1, 60)_ = 9.52,  *p*<0.01 |  |
| S1J | 3-way  ANOVA | Diet:  *F*_(1, 60)_ = 27.05, *p*<0.0001  Hormone:  *F*_(1, 60)_ = 6.86,  *p*<0.05  *APOE* X Diet:  *F*_(2, 60)_ = 3.60,  *p*<0.05 | *APOE3/4*:Control Estradiol vs. *APOE3/4*:HFD Vehicle;  *p*<0.01  *APOE4/4*:Control Estradiol vs. *APOE4/4*:HFD Estradiol;  *p*<0.05  *APOE4/4*:Control Estradiol vs. *APOE4/4*:HFD Vehicle;  *p*<0.01 |
| S1K | 3-way  ANOVA | Diet:  *F*_(1, 60)_ = 5.60,  *p*<0.05  *APOE* X Diet:  *F*_(2, 60)_ = 3.29,  *p*<0.05 |  |
| S1L | 3-way  ANOVA | Diet:  *F*_(1, 60)_ = 15.26,  *p* <0.001  *APOE* X Hormone:  *F*_(2, 60)_ = 4.15,  *p*<0.05  *APOE* X Diet X Hormone:  *F*_(2, 60)_ = 4.29,  *p*<0.05 | *APOE3/4*:Control Vehicle vs. *APOE3/4*:HFD Vehicle;  *p*<0.05  *APOE3/4*:HFD Vehicle vs. *APOE4/4*:Control Vehicle;  *p*<0.01  *APOE4/4*:Control Vehicle vs. *APOE4/4*:HFD Estradiol;  *p*<0.01 |
| S1M | 3-way  ANOVA | Diet:  *F*_(1, 60)_ = 17.40, *p*<0.0001 | *APOE4/4*:Control Vehicle vs. *APOE4/4*:HFD Estradiol;  *p*<0.05  *APOE4/4*:Control Estradiol vs. *APOE4/4*:HFD Estradiol;  *p*<0.05 |
| S1N | 3-way  ANOVA | NS |  |

Abbreviations: 17βE2, 17β-estradiol; HFD, high-fat diet; min, minutes; NS, not significant; RM, repeated measures.

**Table 2.** Summary of statistical analyses with comparisons within *APOE* genotypes.

| **Figure** | **Measure used** | **Genotype** | **Main effects and interactions** | **Selected post hoc comparisons** |
| --- | --- | --- | --- | --- |
| 2D | Two-tailed t-test | *APOE3/3* | p<0.0001; t= 5.4; df= 42 |  |
|  |  | *APOE3/4* | p<0.0001; t= 5.7; df= 42 |  |
|  |  | *APOE4/4* | p<0.0001; t= 6.9; df= 42 |  |
| 2E | 2-way ANOVA | *APOE3/3* | Interaction: F(1, 40)= 26.6; p<0.0001  Hormone: F(1, 40)= 18.1; p<0.0001  Diet: F(1, 40)= 3.4; p=0.07 | Control Vehicle vs. HFD Vehicle; p<0.0001  HFD Vehicle vs. HFD Estradiol; p<0.0001 |
|  |  | *APOE3/4* | Interaction: F(1, 40)= 4.9; p<0.05  Hormone: F(1, 40)= 8.9; p<0.01 | HFD Vehicle vs. HFD Estradiol; p<0.01  Control Vehicle vs. HFD Vehicle; p=0.07 |
|  |  | *APOE4/4* | Interaction: F(1, 40)= 11.9; p<0.01  Hormone: F(1, 40)= 38.8; p<0.0001 | Control Vehicle vs. HFD Vehicle; p<0.05  HFD Vehicle vs. HFD Estradiol; p<0.0001 |
| 2F | 2-way ANOVA | *APOE3/3* | Interaction: F(1, 39)= 9.7; p<0.01  Diet: F(1, 39)= 21.2; p<0.0001  Hormone: F(1, 39)= 21.6; p<0.0001 | Control Vehicle vs. HFD Vehicle; p<0.0001  HFD Vehicle vs. HFD Estradiol; p<0.0001 |
|  |  | *APOE3/4* | Diet: F(1, 39)= 4.3; p<0.05  Hormone: F(1, 39)= 10.1; p<0.01  Interaction: F(1, 39)= 3.0; p=0.09 | HFD Vehicle vs. HFD Estradiol; p<0.01 |
|  |  | *APOE4/4* | Interaction: F(1, 38)= 12.5; p<0.01  Diet: F(1, 38)= 15.8; p<0.001  Hormone: F(1, 38)= 31.4; p<0.0001 | Control Vehicle vs. HFD Vehicle; p<0.0001  HFD Vehicle vs. HFD Estradiol; p<0.0001 |
| 2G | 2-way ANOVA | *APOE3/3* | Interaction: F(1, 35)= 4.9; p<0.05  Diet: F(1, 35)= 11.9; p<0.01  Hormone: F(1, 35)= 30.6; p<0.0001 | Control Vehicle vs. Control Estradiol; p<0.0001  Control Vehicle vs. HFD Vehicle; p<0.01  HFD Vehicle vs. HFD Estradiol; p=0.10 |
|  |  | *APOE3/4* | Diet: F(1, 35)= 9.9; p<0.01  Hormone: F(1, 35)= 41.9; p<0.0001 | Control Vehicle vs. Control Estradiol; p<0.001  HFD Vehicle vs. HFD Estradiol; p<0.001 |
|  |  | *APOE4/4* | Hormone: F(1, 35)= 36.7; p<0.0001 | Control Vehicle vs. Control Estradiol; p<0.01  HFD Vehicle vs. HFD Estradiol; p<0.001 |
| 2H | 2-way ANOVA | *APOE3/3* | Interaction: F(1, 40)= 20.5; p<0.0001  Diet: F(1, 40)= 57.6; p<0.0001  Hormone: F(1, 40)= 58.2; p<0.0001 | Control Vehicle vs. HFD Vehicle; p<0.0001  HFD Vehicle vs. HFD Estradiol; p<0.0001 |
|  |  | *APOE3/4* | Interaction: F(1, 40)= 7.7; p<0.01  Diet: F(1, 40)= 15.7; p<0.001  Hormone: F(1, 40)= 36.0; p<0.0001 | Control Vehicle vs. HFD Vehicle; p<0.001  HFD Vehicle vs. HFD Estradiol; p<0.0001 |
|  |  | *APOE4/4* | Interaction: F(1, 40)= 19.0; p<0.0001  Diet: F(1, 40)= 28.1; p<0.0001  Hormone: F(1, 40)= 75.7; p<0.0001 | Control Vehicle vs. Control Estradiol; p<0.05  Control Vehicle vs. HFD Vehicle; p<0.0001  HFD Vehicle vs. HFD Estradiol; p<0.0001 |
| 2I | 2-way ANOVA | *APOE3/3* | Interaction: F(1, 40)= 16.2; p<0.001  Diet: F(1, 40)= 77.8; p<0.0001  Hormone: F(1, 40)= 50.0; p<0.0001 | Control Vehicle vs. HFD Vehicle; p<0.0001  Control Estradiol vs. HFD Estradiol; p<0.01  HFD Vehicle vs. HFD Estradiol; p<0.0001 |
|  |  | *APOE3/4* | Interaction: F(1, 40)= 6.5; p<0.05  Diet: F(1, 40)= 22.0; p<0.0001  Hormone: F(1, 40)= 34.7; p<0.0001 | Control Vehicle vs. HFD Vehicle; p<0.0001  HFD Vehicle vs. HFD Estradiol; p<0.0001  Control Vehicle vs. Control Estradiol; p=0.10 |
|  |  | *APOE4/4* | Interaction: F(1, 40)= 23.2; p<0.0001  Diet: F(1, 40)= 48.1; p<0.0001  Hormone: F(1, 40)= 90.2; p<0.0001 | Control Vehicle vs. Control Estradiol; p<0.05  Control Vehicle vs. HFD Vehicle; p<0.0001  HFD Vehicle vs. HFD Estradiol; p<0.0001 |
| 3D | 2-way ANOVA | *APOE3/3* | Interaction: F(1, 40)= 11.2; p<0.01 | Control Vehicle vs. HFD Vehicle; p<0.01  HFD Vehicle vs. HFD Estradiol; p<0.05 |
|  |  | *APOE3/4* | No significant differences |  |
|  |  | *APOE4/4* | Diet: F(1, 40)= 7.6; p<0.01  Hormone: F(1, 40)= 3.9; p=0.054 | Control Vehicle vs. HFD Vehicle; p<0.05 |
| 3E | 2-way ANOVA | *APOE3/3* | Interaction: F(1, 40)= 9.3; p<0.01  Diet: F(1, 40)= 27.6; p<0.0001 | Control Vehicle vs. Control Estradiol; p<0.05  Control Vehicle vs. HFD Vehicle; p<0.0001 |
|  |  | *APOE3/4* | Diet: F(1, 40)= 33.6; p<0.0001 | Control Vehicle vs. HFD Vehicle; p<0.001  Control Estradiol vs. HFD Estradiol; p<0.01 |
|  |  | *APOE4/4* | Diet: F(1, 40)= 16.4; p<0.001  Hormone: F(1, 40)= 2.8; p=0.10 | Control Vehicle vs. HFD Vehicle; p<0.01 |
| 3F | 2-way ANOVA | *APOE3/3* | Hormone: F(1, 35)= 5.6; p<0.05 |  |
|  |  | *APOE3/4* | Hormone: F(1, 34)= 5.8; p<0.05 |  |
|  |  | *APOE4/4* | Hormone: F(1, 35)= 12.5; p<0.01 | HFD Vehicle vs. HFD Estradiol; p<0.01 |
| 5A | 2-way ANOVA | *APOE3/3* | Hormone: F(1, 39)= 6.3; p<0.05  Diet: F(1, 39)= 3.1; p=0.09 | HFD Vehicle vs. HFD Estradiol; p<0.05 |
|  |  | *APOE3/4* | No significant differences |  |
|  |  | *APOE4/4* | Hormone: F(1, 39)= 7.9; p<0.01 | HFD Vehicle vs. HFD Estradiol; p=0.052 |
| 5B | 2-way ANOVA | *APOE3/3* | Diet: F(1, 37)= 4.1; p<0.05 |  |
|  |  | *APOE3/4* | No significant differences |  |
|  |  | *APOE4/4* | Hormone: F(1, 35)= 10.7; p<0.01 | Control Vehicle vs. Control Estradiol; p<0.05 |
| 5C | 2-way ANOVA | *APOE3/3* | No significant differences |  |
|  |  | *APOE3/4* | No significant differences |  |
|  |  | *APOE4/4* | Hormone: F(1, 31)= 8.0; p<0.01 | HFD Vehicle vs. HFD Estradiol; p=0.08 |
| 5G | 2-way ANOVA | *APOE3/3* | No significant differences |  |
|  |  | *APOE3/4* | No significant differences |  |
|  |  | *APOE4/4* | No significant differences |  |
| 6D | 2-way ANOVA | *APOE3/3* | No significant differences |  |
|  |  | *APOE3/4* | Hormone: F(1, 40)= 4.7; p<0.05 |  |
|  |  | *APOE4/4* | Hormone: F(1, 40)= 5.1; p<0.05  Diet: F(1, 40)= 3.1; p=0.08 |  |
| 6E | 2-way ANOVA | *APOE3/3* | Interaction: F(1, 39)= 14.8; p<0.001  Diet: F(1, 39)= 28.4; p<0.0001  Hormone: F(1, 39)= 16.5; p<0.001 | Control Vehicle vs. HFD Vehicle; p<0.0001  HFD Vehicle vs. HFD Estradiol; p<0.0001 |
|  |  | *APOE3/4* | Interaction: F(1, 40)= 4.2; p<0.05  Hormone: F(1, 40)= 5.1; p<0.05  Diet: F(1, 40)= 3.6; p=0.06 | Control Vehicle vs. HFD Vehicle; p<0.05  HFD Vehicle vs. HFD Estradiol; p<0.05 |
|  |  | *APOE4/4* | Hormone: F(1, 40)= 6.2; p<0.05  Interaction: F(1, 40)= 3.6; p=0.06 | HFD Vehicle vs. HFD Estradiol; p<0.05 |
| S1A | 2-way ANOVA | *APOE3/3* | Diet: F(1, 20)= 5.4; p<0.05 |  |
|  |  | *APOE3/4* | No significant differences |  |
|  |  | *APOE4/4* | Diet: F(1, 20)= 13.5; p<0.01 | Control Estradiol vs. HFD Estradiol; p<0.05 |
| S1B | 2-way ANOVA | *APOE3/3* | No significant differences |  |
|  |  | *APOE3/4* | Interaction: F(1, 20)= 11.9; p<0.01  Hormone: F(1, 20)= 13.2; p<0.01  Diet: F(1, 20)= 4.3; p=0.05 | Control Vehicle vs. HFD Vehicle; p<0.01  HFD Vehicle vs. HFD Estradiol; p<0.001 |
|  |  | *APOE4/4* | Diet: F(1, 20)= 22.9; p<0.001  Hormone: F(1, 20)= 5.6; p<0.05 | Control Vehicle vs. HFD Vehicle; p<0.05  Control Estradiol vs. HFD Estradiol; p<0.05 |
| S1C | 2-way ANOVA | *APOE3/3* | No significant differences |  |
|  |  | *APOE3/4* | Interaction: F(1, 20)= 5.5; p<0.05  Diet: F(1, 20)= 4.4; p=0.05 | Control Vehicle vs. HFD Vehicle; p<0.05  HFD Vehicle vs. HFD Estradiol; p=0.06 |
|  |  | *APOE4/4* | Diet: F(1, 20)= 8.6; p<0.01  Hormone: F(1, 20)= 10.6; p<0.01  Interaction: F(1, 20)= 3.5; p=0.07 | Control Estradiol vs. HFD Estradiol; p<0.05  HFD Vehicle vs. HFD Estradiol; p<0.01 |
| S1D | 2-way ANOVA | *APOE3/3* | Diet: F(1, 20)= 4.5; p<0.05 |  |
|  |  | *APOE3/4* | Interaction: F(1, 20)= 4.5; p<0.05  Diet: F(1, 20)= 8.3; p<0.01  Hormone: F(1, 20)= 5.4; p<0.05 | Control Vehicle vs. HFD Vehicle; p<0.05  HFD Vehicle vs. HFD Estradiol; p<0.05 |
|  |  | *APOE4/4* | Interaction: F(1, 20)= 9.0; p<0.01  Diet: F(1, 20)= 12.8; p<0.01 | Control Estradiol vs. HFD Estradiol; p<0.001  HFD Vehicle vs. HFD Estradiol; p<0.05 |
| S1E | 2-way ANOVA | *APOE3/3* | No significant differences |  |
|  |  | *APOE3/4* | No significant differences |  |
|  |  | *APOE4/4* | Diet: F(1, 20)= 15.7; p<0.001 | Control Estradiol vs. HFD Estradiol; p<0.01 |
| S1F | 2-way ANOVA | *APOE3/3* | Hormone: F(1, 20)= 6.6; p<0.05 |  |
|  |  | *APOE3/4* | Interaction: F(1, 20)= 4.4; p<0.05  Hormone: F(1, 20)= 13.8; p<0.01 | HFD Vehicle vs. HFD Estradiol; p<0.01 |
|  |  | *APOE4/4* | Diet: F(1, 20)= 5.3; p<0.05 |  |
| S1G | 2-way ANOVA | *APOE3/3* | No significant differences |  |
|  |  | *APOE3/4* | Interaction: F(1, 20)= 5.3; p<0.05  Hormone: F(1, 20)= 3.2; p=0.09 | HFD Vehicle vs. HFD Estradiol; p<0.05 |
|  |  | *APOE4/4* | Diet: F(1, 20)= 4.4; p<0.05  Hormone: F(1, 20)= 3.0; p=0.09 | Control Estradiol vs. HFD Estradiol; p=0.07 |
| S1H | 2-way ANOVA | *APOE3/3* | No significant differences |  |
|  |  | *APOE3/4* | No significant differences |  |
|  |  | *APOE4/4* | Hormone: F(1, 20)= 9.8; p<0.01 | Control Vehicle vs. Control Estradiol; p=0.07 |
| S1I | 2-way ANOVA | *APOE3/3* | No significant differences |  |
|  |  | *APOE3/4* | Interaction: F(1, 20)= 4.7; p<0.05 | Control Vehicle vs. HFD Vehicle; p=0.10 |
|  |  | *APOE4/4* | Diet: F(1, 20)= 12.6; p<0.01 | Control Estradiol vs. HFD Estradiol; p<0.05 |
| S1J | 2-way ANOVA | *APOE3/3* | No significant differences |  |
|  |  | *APOE3/4* | Diet: F(1, 20)= 15.8; p<0.001  Hormone: F(1, 20)= 3.5; p=0.08 | Control Estradiol vs. HFD Estradiol; p<0.05  Control Vehicle vs. HFD Vehicle; p=0.10 |
|  |  | *APOE4/4* | Diet: F(1, 20)= 14.9; p<0.01 | Control Estradiol vs. HFD Estradiol; p<0.05 |
| S1K | 2-way ANOVA | *APOE3/3* | No significant differences |  |
|  |  | *APOE3/4* | No significant differences |  |
|  |  | *APOE4/4* | Diet: F(1, 20)= 12.0; p<0.01 | Control Estradiol vs. HFD Estradiol; p<0.05 |
| S1L | 2-way ANOVA | *APOE3/3* | No significant differences |  |
|  |  | *APOE3/4* | Interaction: F(1, 20)= 8.0; p<0.05  Diet: F(1, 20)= 4.9; p<0.05 | Control Vehicle vs. HFD Vehicle; p<0.01  HFD Vehicle vs. HFD Estradiol; p=0.052 |
|  |  | *APOE4/4* | Diet: F(1, 20)= 12.0; p<0.01  Hormone: F(1, 20)= 8.0; p<0.05 | Control Estradiol vs. HFD Estradiol; p<0.05  HFD Vehicle vs. HFD Estradiol; p<0.05 |
| S1M | 2-way ANOVA | *APOE3/3* | No significant differences |  |
|  |  | *APOE3/4* | Diet: F(1, 20)= 4.6; p<0.05  Hormone: F(1, 20)= 4.6; p<0.05 |  |
|  |  | *APOE4/4* | Diet: F(1, 20)= 14.0; p<0.01 | Control Estradiol vs. HFD Estradiol; p<0.05 |
| S1N | 2-way ANOVA | *APOE3/3* | No significant differences |  |
|  |  | *APOE3/4* | No significant differences |  |
|  |  | *APOE4/4* | No significant differences |  |

Abbreviations: HFD, high-fat diet.

**Supplemental Figure 1. Effects of *APOE* genotype, HFD, and estradiol on plasma lipid classes.** Plasma samples collected at Week 16 were assessed by shotgun lipidomics. Data show normalized class abundance for (A) Fatty acids, (B) ceramides, (C) phosphatidylcholine, (D) phosphatidylinositol, (E) phosphatidylethanolamine, (F) hexosyl ceramides, (G) triglycerides, (H) phosphatidylglycerol, (I) cholesterol esters, (J) diacylglycerols, (K) phosphatidic acid, (L) lyso-phosphatidylethanolamine, (M) lyso-phosphatidylcholine, and (N) sphingomyelin. Bars colored green are used for *APOE3/3* mice, blue for *APOE3/4* mice, and red for *APOE4/4* mice. Lighter shades represent Control diet, darker shades represent HFD. Solid bars represent vehicle capsules, hatched bars represent 17β-estradiol capsules. All data are represented as the mean ± SEM; n= 6 per group. Statistical significance with *p*<0.05 is denoted by asterisks. Brackets indicate significant differences between specified groups.


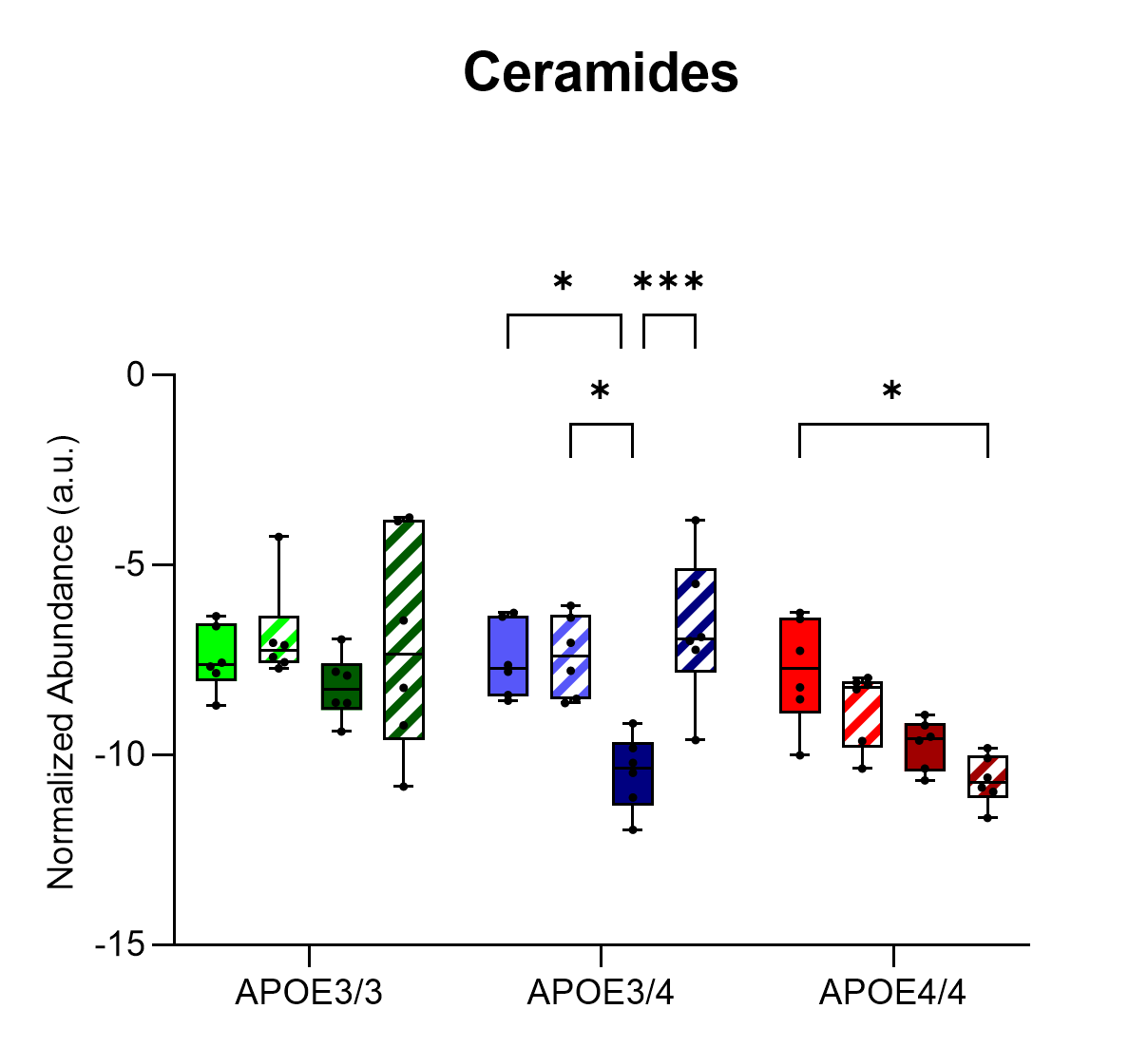

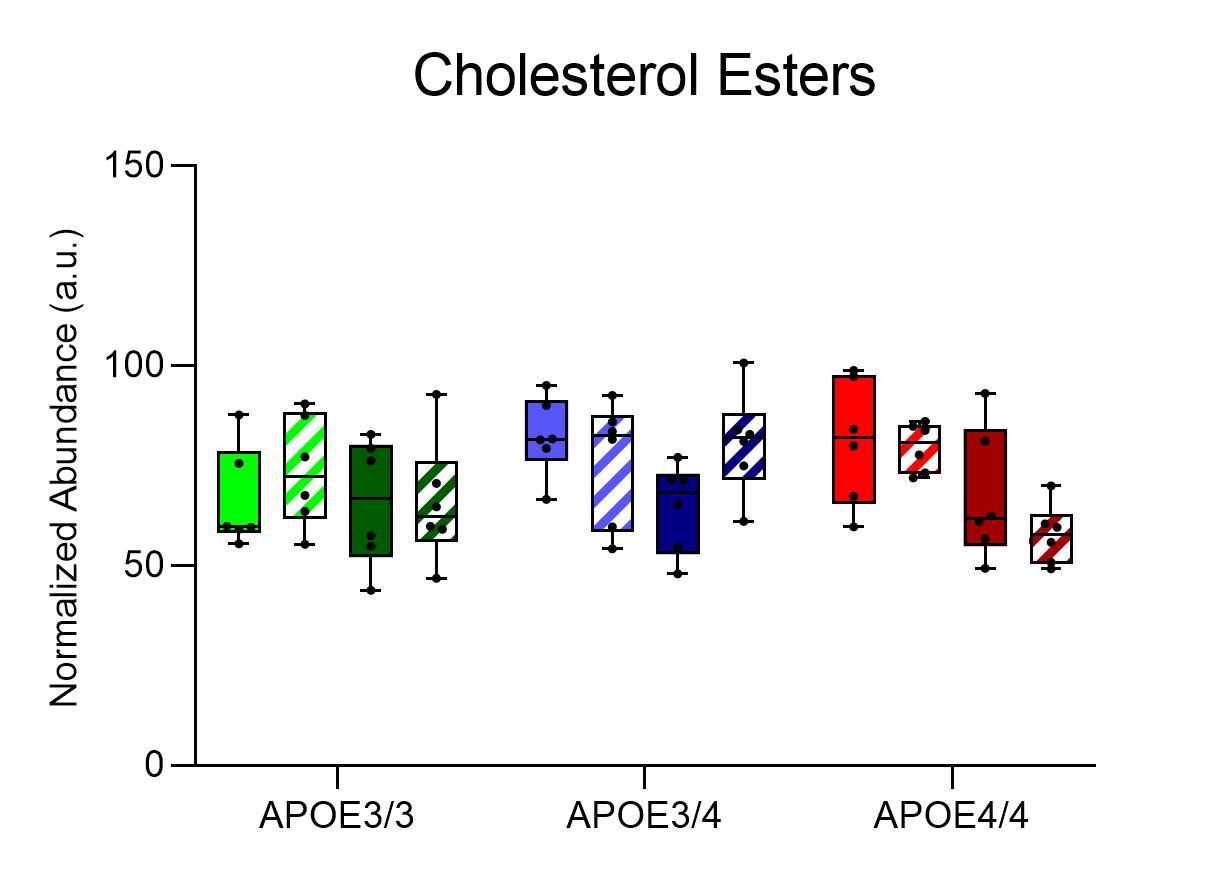

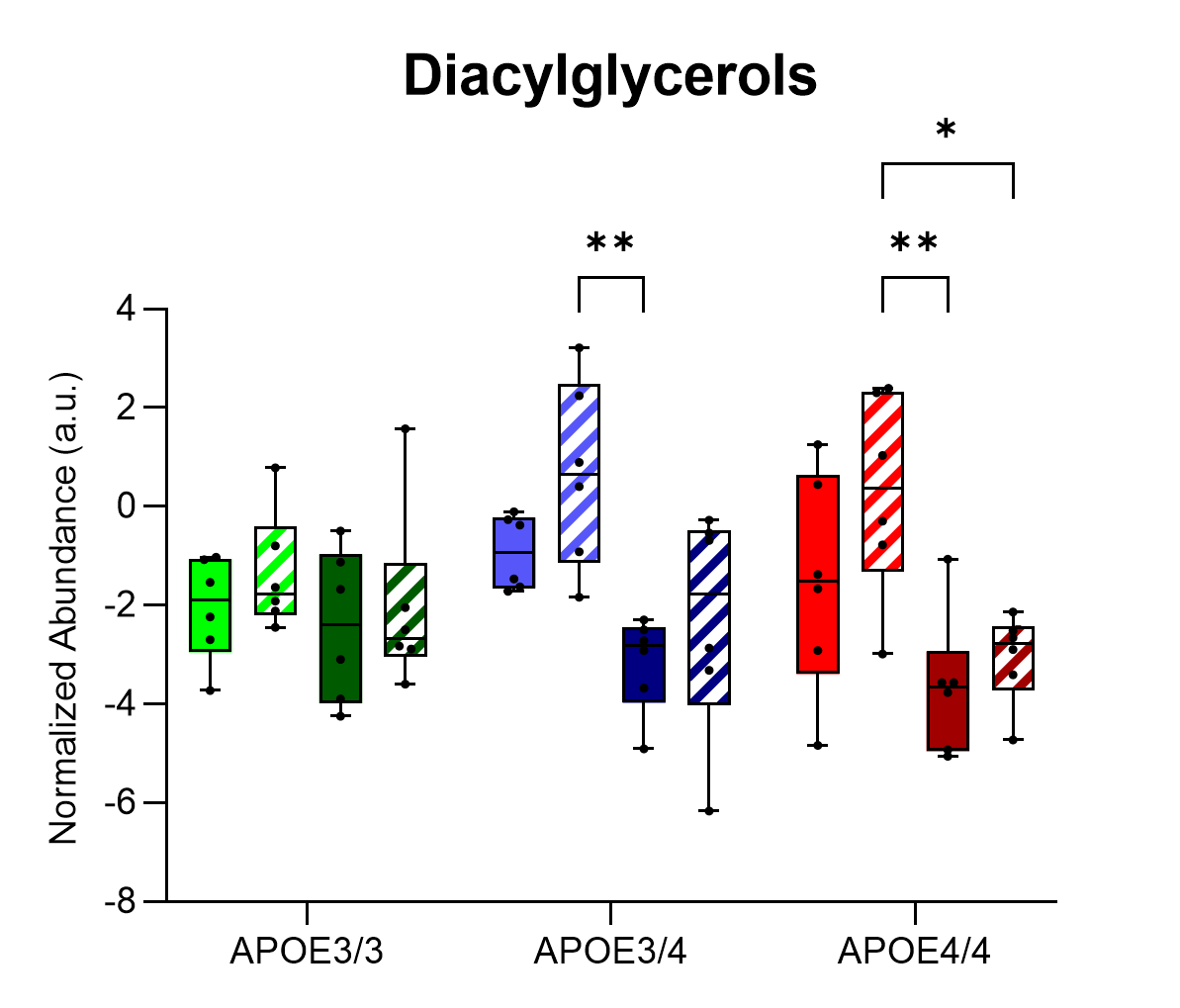

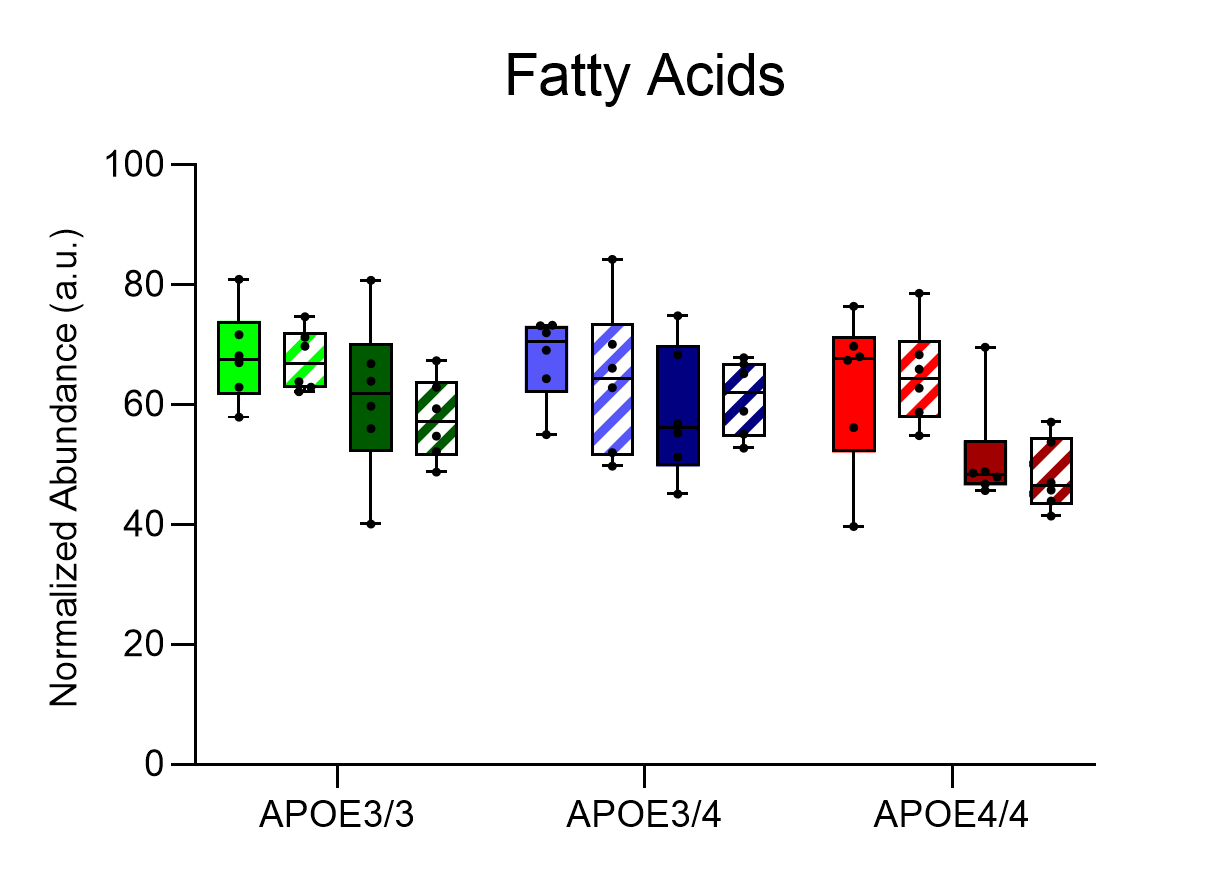

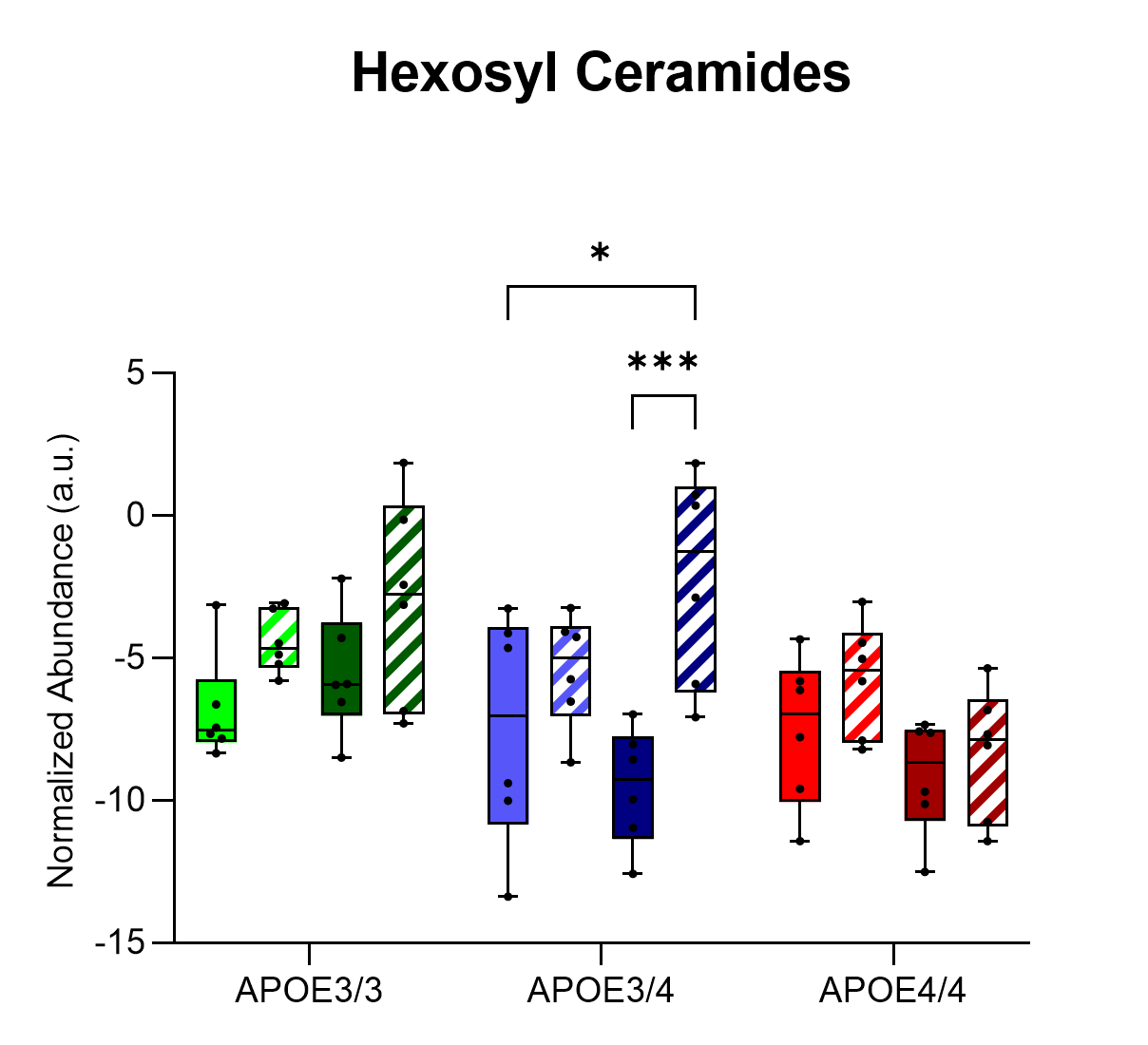

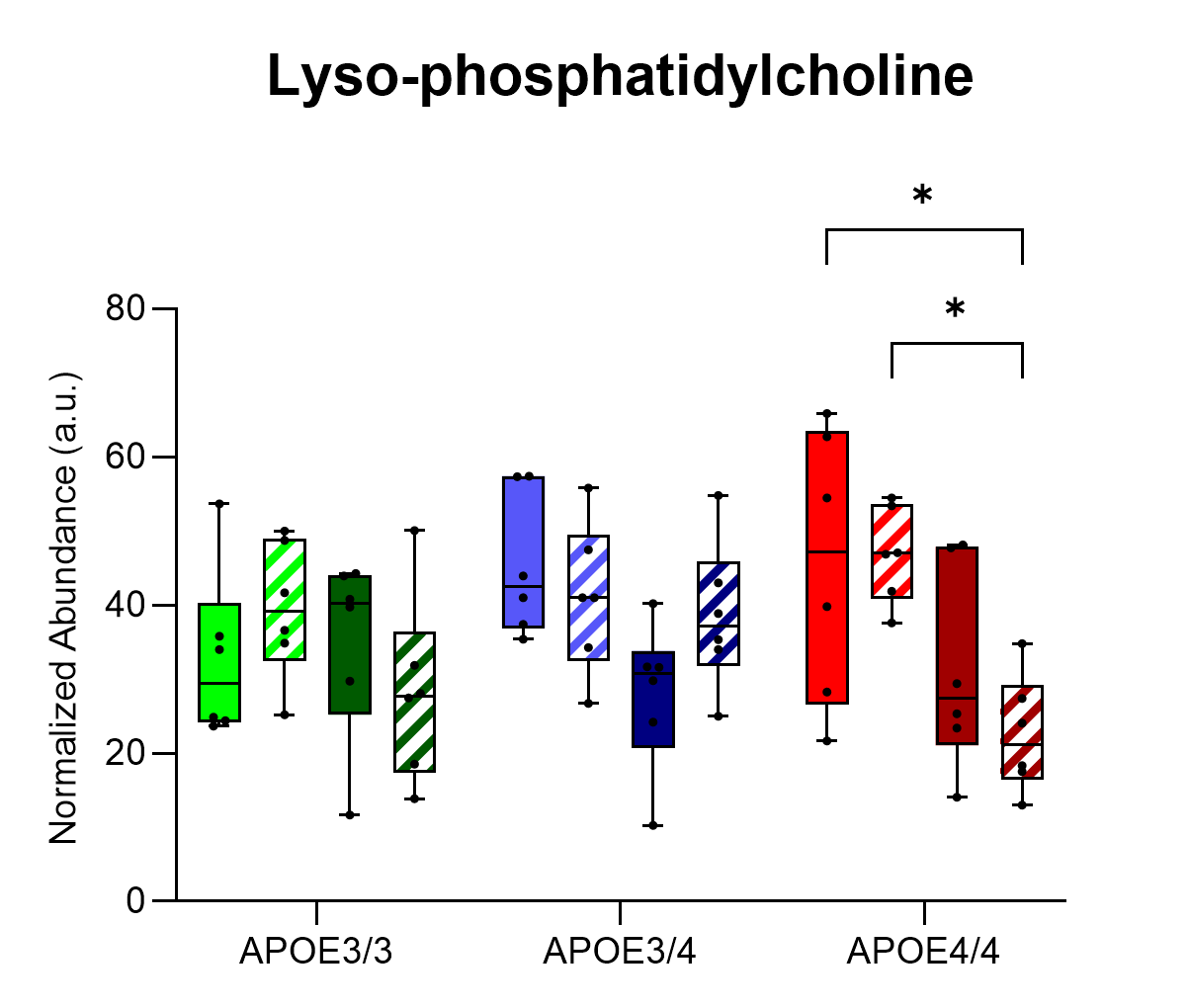

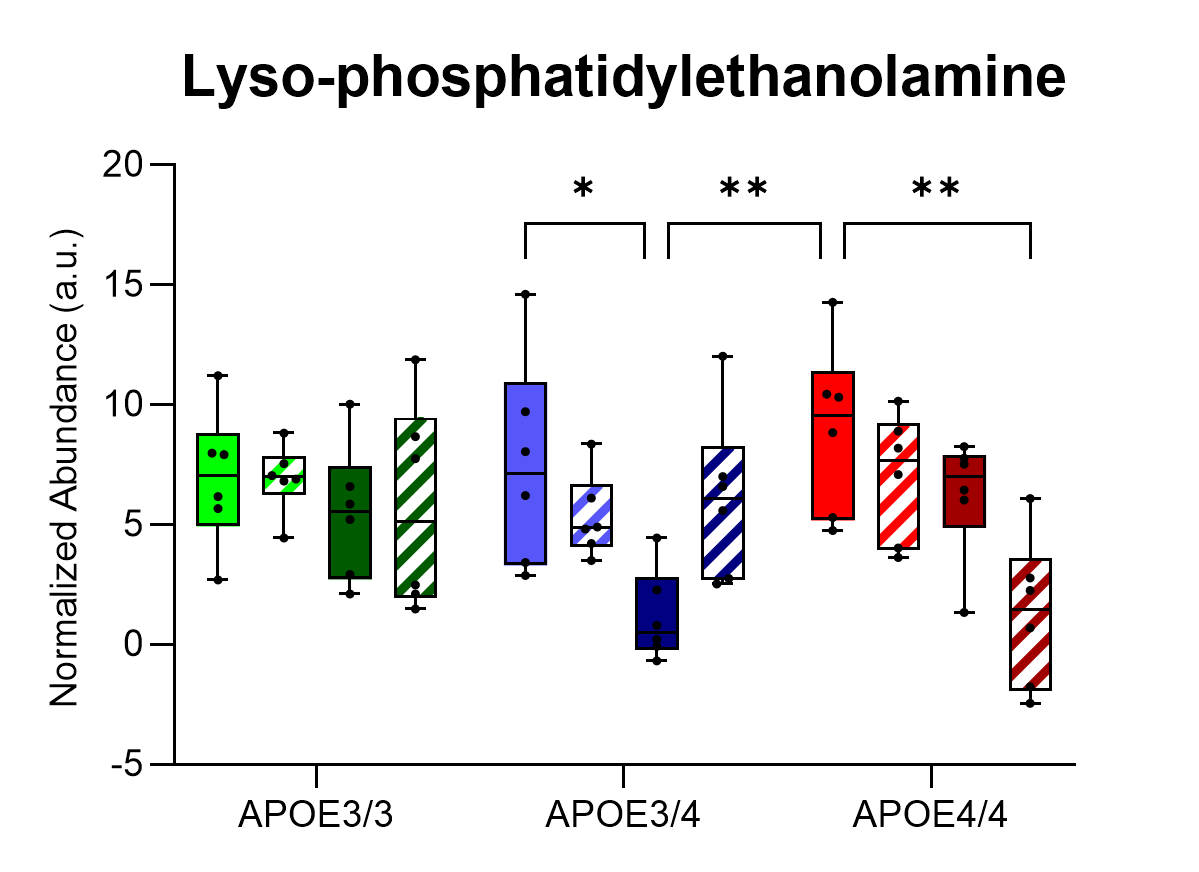

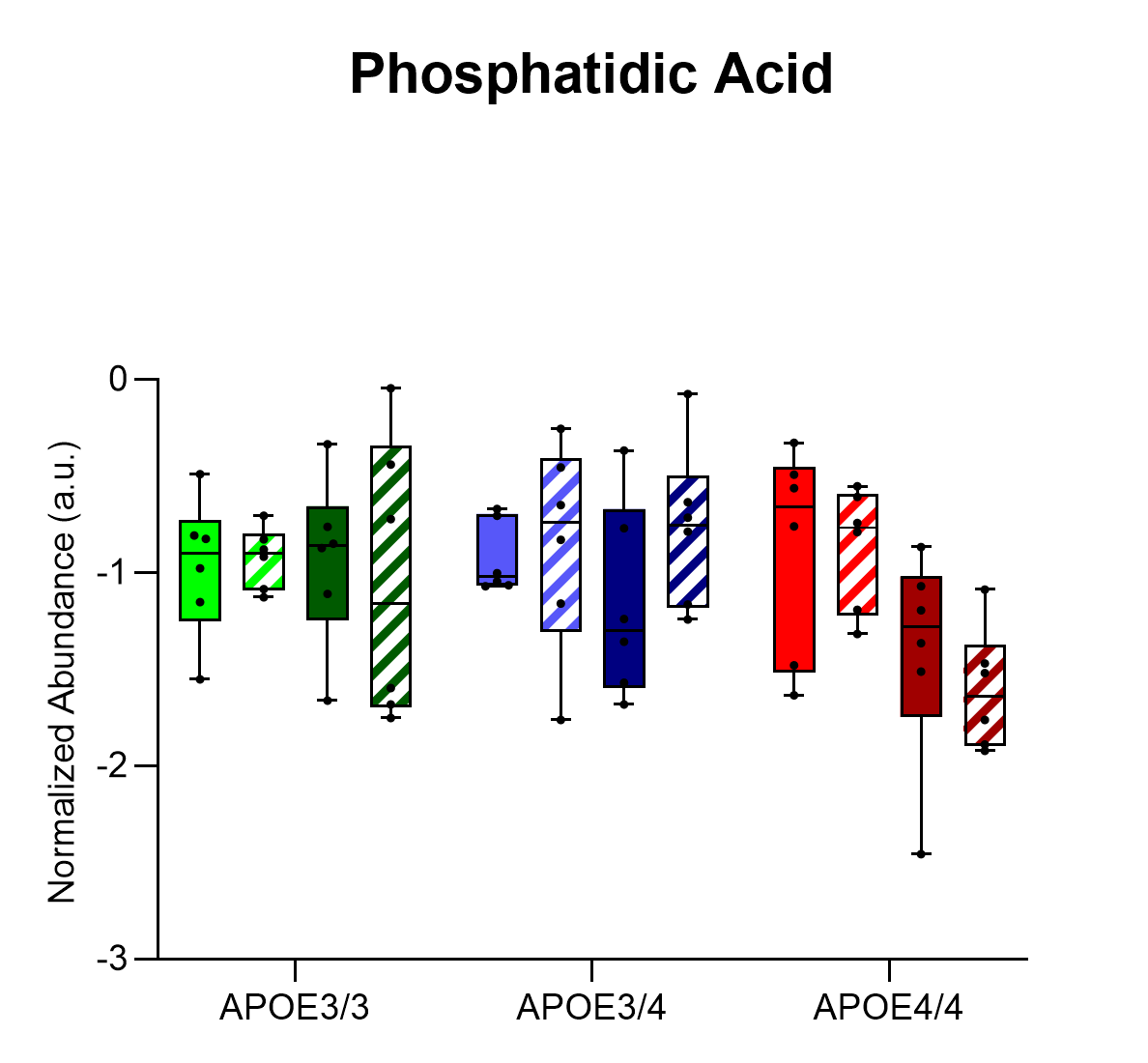

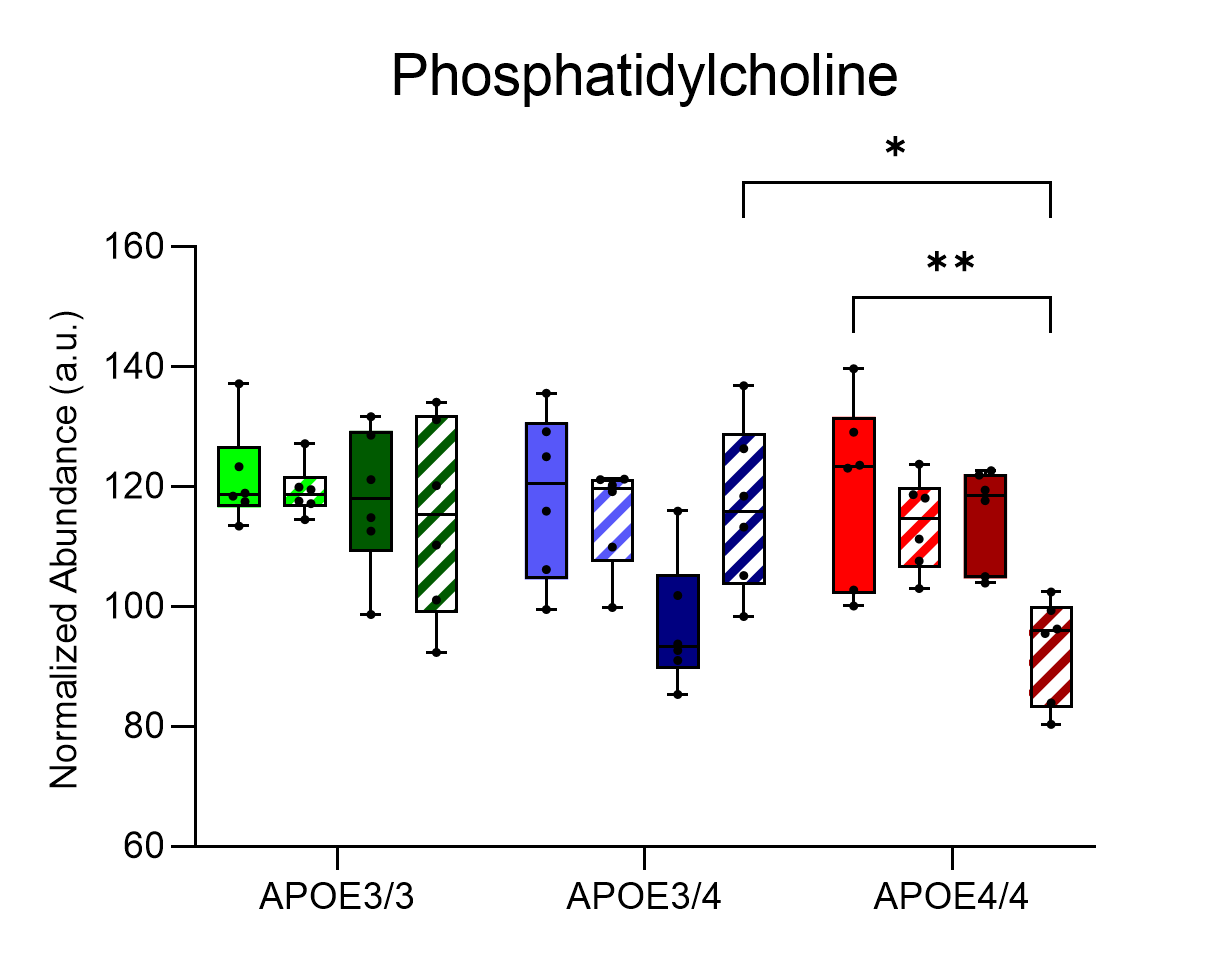

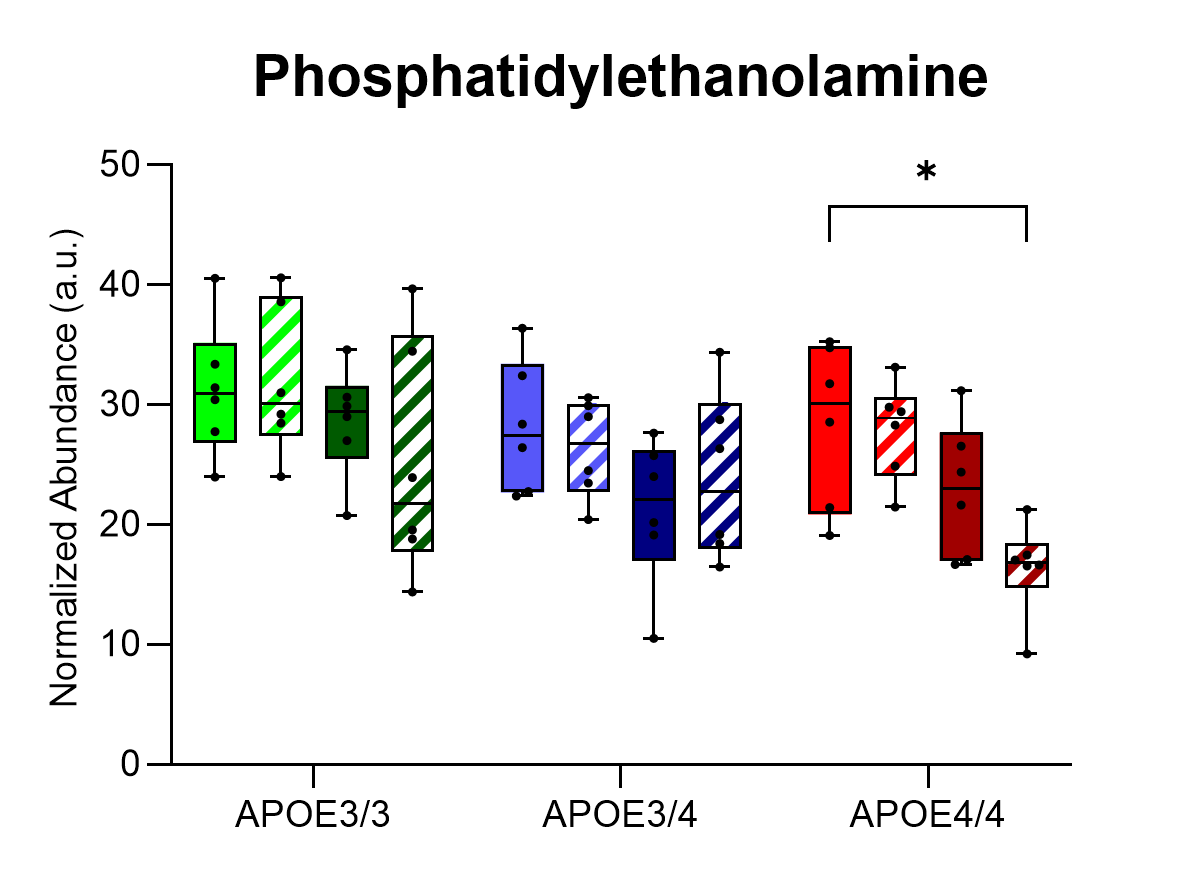

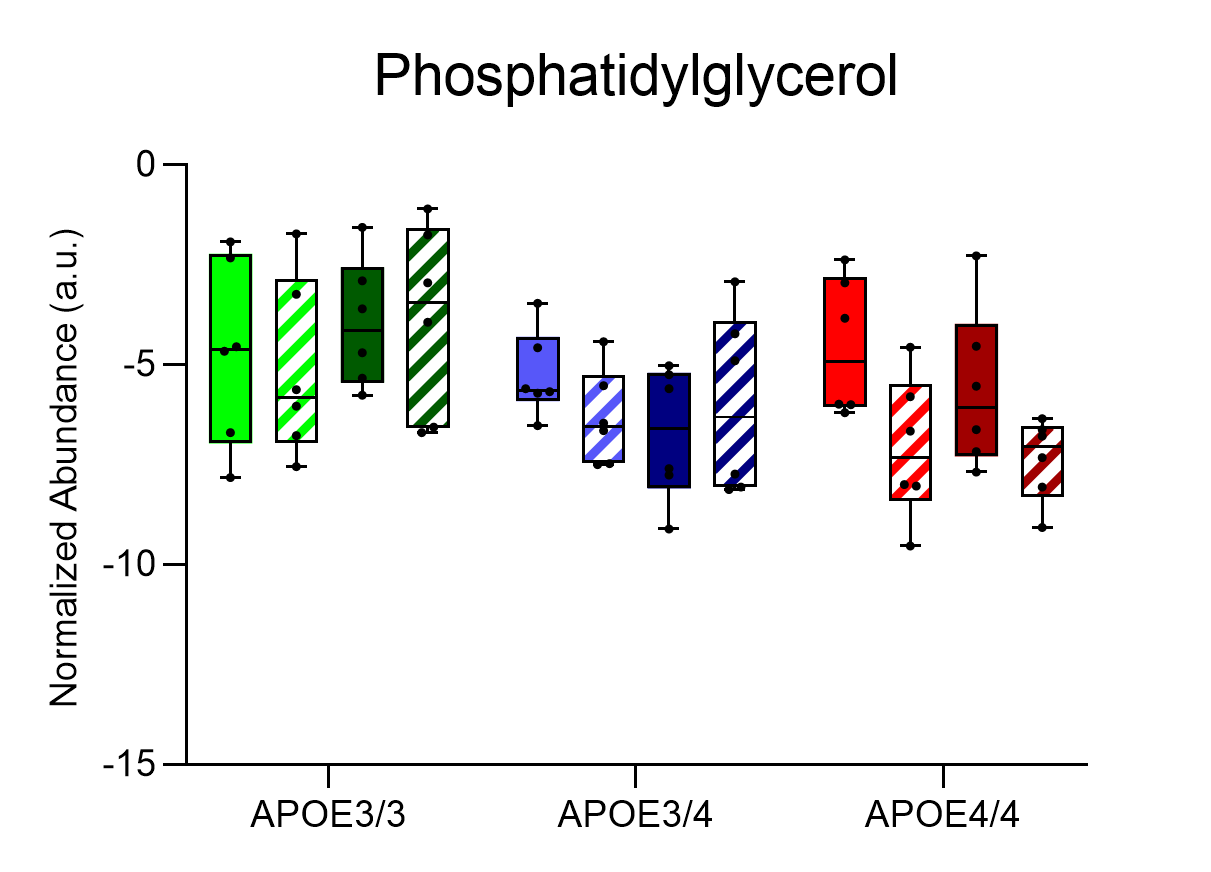

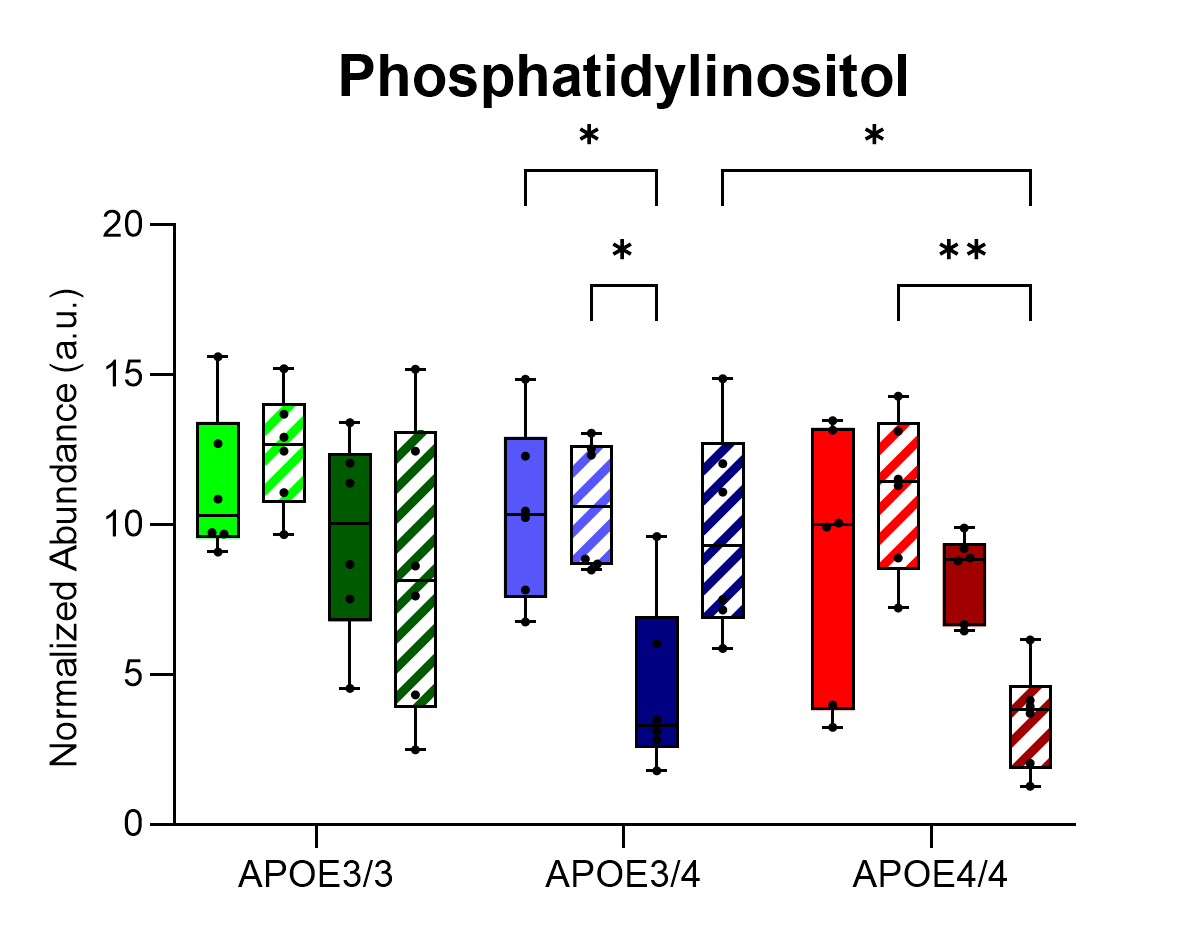

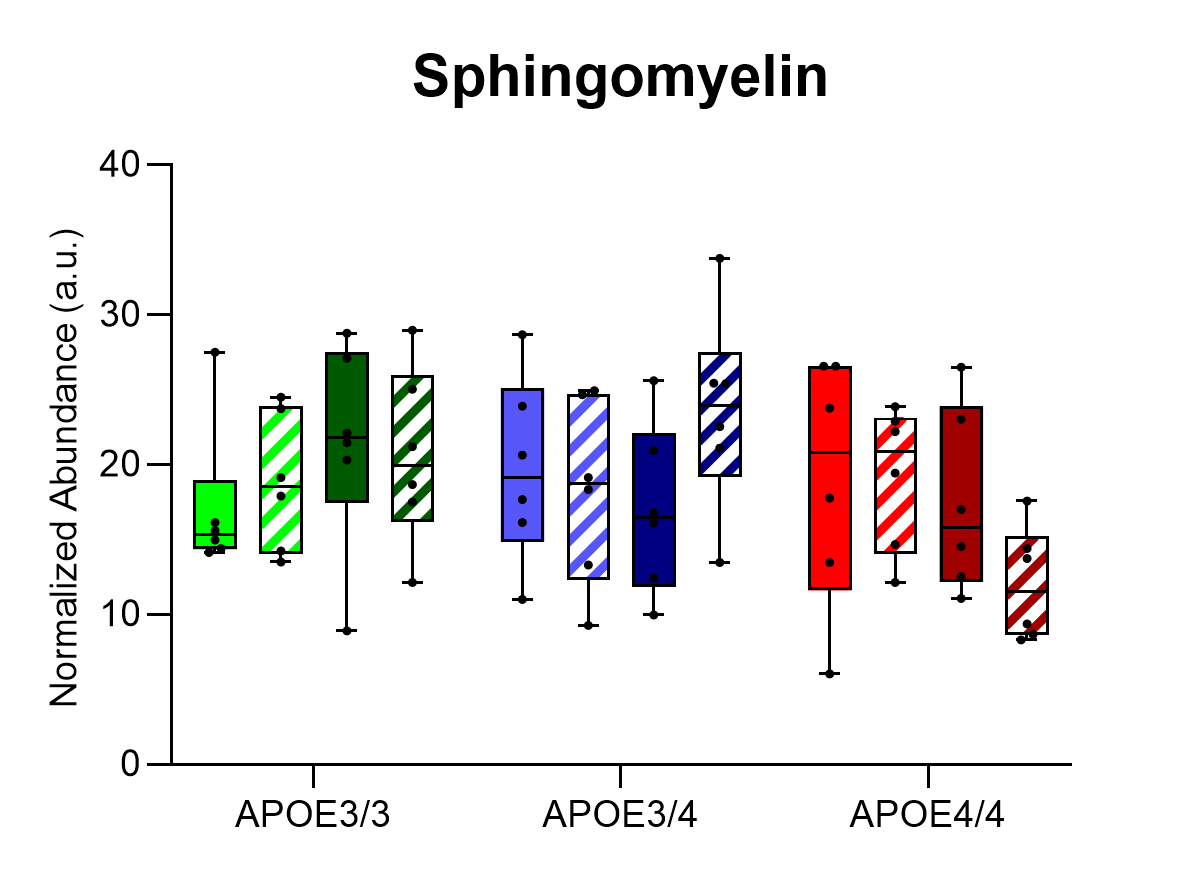

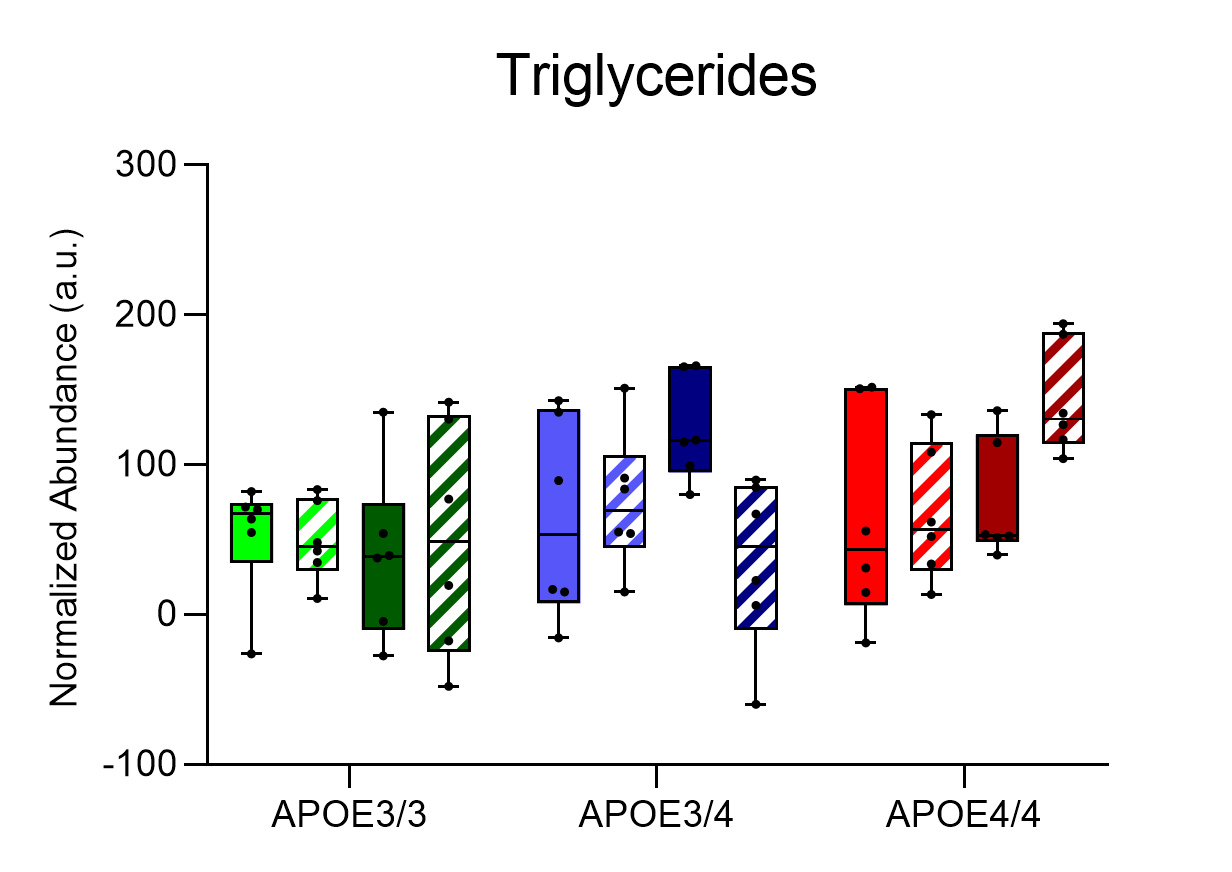


**Fatty Acids**

**Ceramides**

**Phosphatidylcholine**

**Phosphatidylinositol**

**Phosphatidylethanolamine**

**Hexosyl Ceramides**

**Triglycerides**

**Phosphatidylglycerol**

**Cholesterol Esters**

**Diacylglycerols**

**Phosphatidic Acid**

**Lyso-phosphatidylethanolamine**

**Lyso-phosphatidylcholine**

**Sphingomyelin**

**A**

**L**

**F**

**J**

**K**

**I**

**H**

**G**

**E**

**D**

**C**

**B**

**M**

**N**
